# Kinetic Resolution of Racemic 2-Hydroxyamides Using a Diphenylacetyl Component as an Acyl Source and a Chiral Acyl-Transfer Catalyst

**DOI:** 10.3390/molecules23082003

**Published:** 2018-08-10

**Authors:** Takatsugu Murata, Tatsuya Kawanishi, Akihiro Sekiguchi, Ryo Ishikawa, Keisuke Ono, Kenya Nakata, Isamu Shiina

**Affiliations:** 1 Department of Appliaed Chemistry, Faculty of Science, Tokyo University of Science, Tokyo 162-8601, Japan; b116707@ed.tus.ac.jp (T.M.); tatsuya.kawanishi@tcichemicals.com (T.K.); akihiro.sekiguchi@nipponkayaku.co.jp (A.S.); 12ishikawa25@gmail.com (R.I.); ki_ono@jp.daicel.com (K.O.); 2 Graduate School of Natural Science and Technology, Shimane University, Shimane 690-8504, Japan

**Keywords:** kinetic resolution, 2-hydroxyamide, organocatalysis, Weinreb amide, esterification, carboxylic anhydride

## Abstract

Various optically active 2-hydroxyamide derivatives are produced based on the kinetic resolution of racemic 2-hydroxyamides with a diphenylacetyl component and (*R*)-benzotetramisole ((*R*)-BTM), a chiral acyl-transfer catalyst, via asymmetric esterification and acylation. It was revealed that a tertiary amide can be used with this novel protocol to achieve high selectivity (22 examples; *s*-value reaching over 250). The resulting chiral compounds could be transformed into other useful structures while maintaining their chirality.

## 1. Introduction

**Scheme 1:**
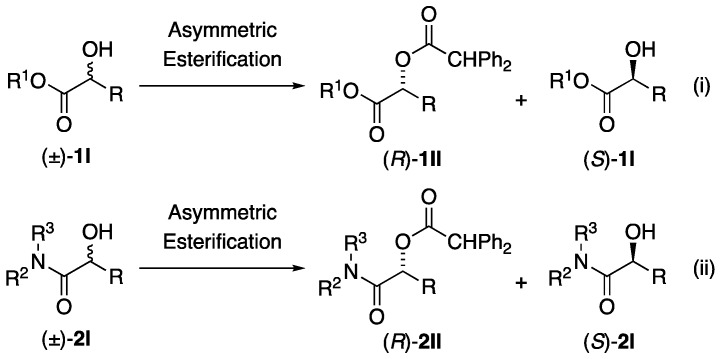
Our previous result (i) and working hypothesis for the present study (ii).

Optically active 2-hydroxyamide derivatives are frequently utilized as chiral building blocks not only for synthesizing biologically active compounds [1,2,3,4], but also for preparing asymmetric catalysts and chiral auxiliaries [5,6]. Consequently, considerable effort has been devoted toward developing efficient methods for synthesizing these compounds, including enzymatic [7] and chemical transformations [8,9,10]. For the purpose of providing chiral alcohols, the kinetic resolution (KR) of racemic alcohols by asymmetric acylation using organocatalysis is widely used as one of the most effective methods [11,12,13]. However, to the best of our knowledge, a general method for the kinetic resolution of racemic 2-hydroxyamide derivatives has not been reported to date. We recently accomplished the first KR of racemic alcohols with achiral carboxylic acids and of racemic carboxylic acids with achiral alcohols by asymmetric esterification [14,15,16,17,18,19,20,21,22,23,24,25,26,27] via the in situ formation of a mixed anhydride using carboxylic anhydrides as coupling reagents combined with chiral acyl-transfer catalysts. Furthermore, KR of racemic 2-hydroxyalkanoates with diphenylacetic acid was achieved using pivalic anhydride in the presence of (*R*)-benzotetramisole [27,28] ((*R*)-BTM; Scheme 1; (i)). Therefore, it was hypothesized that this KR protocol could be similarly applied to 2-hydroxyamide derivatives (Scheme 1; (ii)). In this article, we report the novel KR of various racemic 2-hydroxyamide derivatives using a diphenylacetyl component as an acyl source, catalyzed by (*R*)-BTM.

## 2. Results and Discussion

To determine suitable structures for the amide moiety, the KR reactions of a variety of racemic 2-hydroxyamides were initially examined using diphenylacetyl sources derived from Ph_2_CHCO_2_H or (Ph_2_CHCO)_2_O (DPHAA) [29], catalyzed by (*R*)-BTM in Et_2_O at room temperature for 12 h, which were reaction conditions similar to those established in the previous study (Table 1). We first performed the KR of the secondary *N*-alkyl amide with methyl (±)-**1a** or benzyl (±)-**1b** and *N*-phenyl amide (±)-**1c** via asymmetric esterification. These substrates were found to be unsuitable for the reaction (Entries 1–3). Conversely, it was found that the tertiary amide yielded high *s*-values under the reaction conditions [30]. The KR of (±)-**1d** smoothly proceeded, affording the corresponding ester (*R*)-**2d** (48%; 92% ee) and the recovered alcohol (*S*)-**1d** (46%; >99% ee) with a high *s*-value (Entry 4; *s* = 254). It is noteworthy that *N*-methoxy-*N*-methylamide (±)-**1e** (known as Weinreb amide) [31,32,33] was successfully applied to this protocol with high synthetic utility (Entry 5; *s* = 156). As the tertiary amide was recognized as a suitable structure for attaining high selectivity, we subsequently performed the KR via asymmetric acylation and not via asymmetric esterification for the same reaction. As expected, high selectivity was also achieved by the reaction of (±)-**1d** and **1e** using the asymmetric acylation protocol (Entries 6 and 7).

To assess the generality of this novel method, various racemic 2-hydroxy-*N*,*N*-dimethylamides (±)-**3a**–**3k** with different substituted forms (Table 2) were subjected to asymmetric esterification (condition A1) and asymmetric acylation (condition B1). When the KR of **3a**–**3c**, **3e**, and **3h**, bearing normal aliphatic alkyl chains at the C-2 positions, was performed under the conditions A1 and B1, the reaction successfully proceeded with high *s*-values in all cases. Asymmetric esterification (condition A1) tended to show better results than asymmetric acylation (condition B1); however, it was revealed that the chiral acylation protocol was also useful for obtaining good *s*-values. In contrast, the reaction of (±)-**3d** and **3g**, bearing branched aliphatic alkyl chains (R = *i*-Pr and *c*-Hex) at the C-2 positions, showed a slight decrease in selectivity, while the reaction of **3f** (R = *i*-Bu) yielded a good *s*-value. We also examined several racemic ω-(*tert*-butyldimethylsiloxy)-2-hydroxy-*N*,*N*-dimethylamide derivatives (±)-**3i**–**3k**, having different methylene lengths, as shown in Entries 17–22. It was found that the selectivity of the KR of (±)-**3i** was somewhat lowered by the influence of the siloxy group at the C-3 position (Entries 17 and 18). Other reactions yielded high *s*-values, regardless of the length of the alkyl chains possessing *tert*-butyldimethylsiloxy groups under the conditions A1 and B1 (Entries 19–22).

Furthermore, we performed the KR of various racemic 2-hydroxy-Weinreb amides (±)-**5a**–**5k** with substitution patterns corresponding to the *N*,*N*-dimethylamides (±)-**3a**–**3k** using a similar protocol (Table 3). Consequently, the same tendency was observed. The KR of 2-hydroxy-Weinreb amides **5a**–**5c**, **5e**, **5f**, **5h**, **5j**, and **5k**, bearing normal aliphatic alkyl chains at the C-2 positions, exhibited high *s*-values in all cases under the conditions A1 and B2. Conversely, the reactions of 2-hydroxy-Weinreb amides (±)-**5d**, **5g**, and **5i**, bearing branched aliphatic alkyl chains at the C-2 positions or a siloxy group at the C-3 position, exhibited decreased selectivity.

**Scheme 2:**
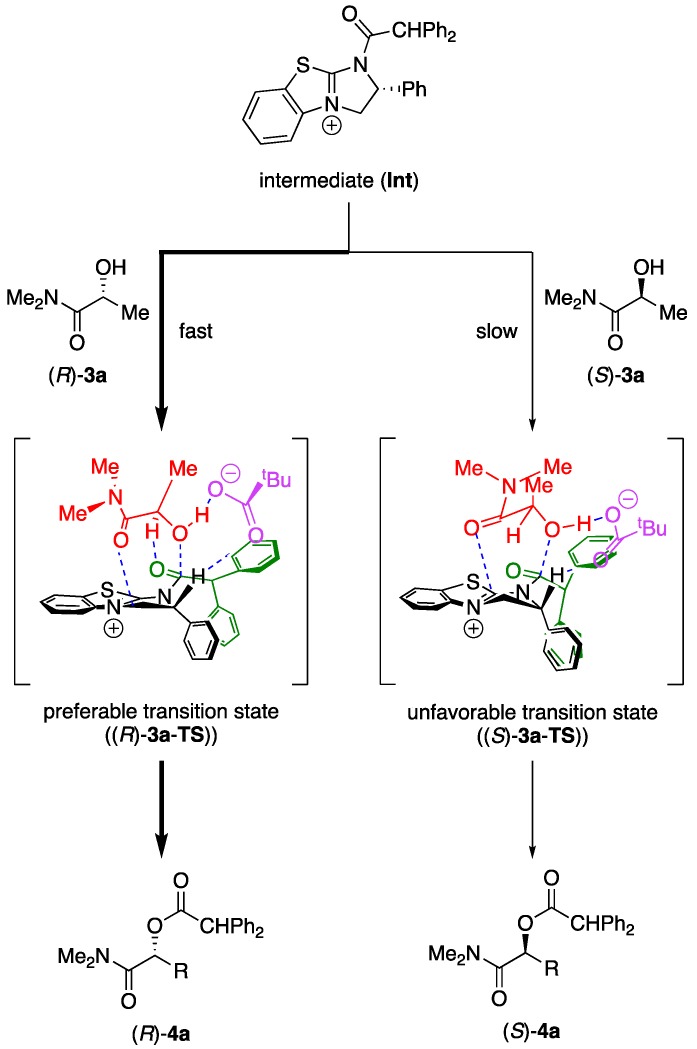
Calculated transition states with a kinetic resolution (KR) of (±)-**3**.

To support the results of the experimental data, we calculated the transition state of each enantiomer in the KR. This was performed using density functional theory (DFT) calculations at the B3LYP/6-31G*//B3LYP/6-31G* level according to a previously reported method [23,27,28]. Initially, we conducted a theoretical study on the KR of 2-hydroxy dimethylamides (Scheme 2) [34].

**Figure 1:**
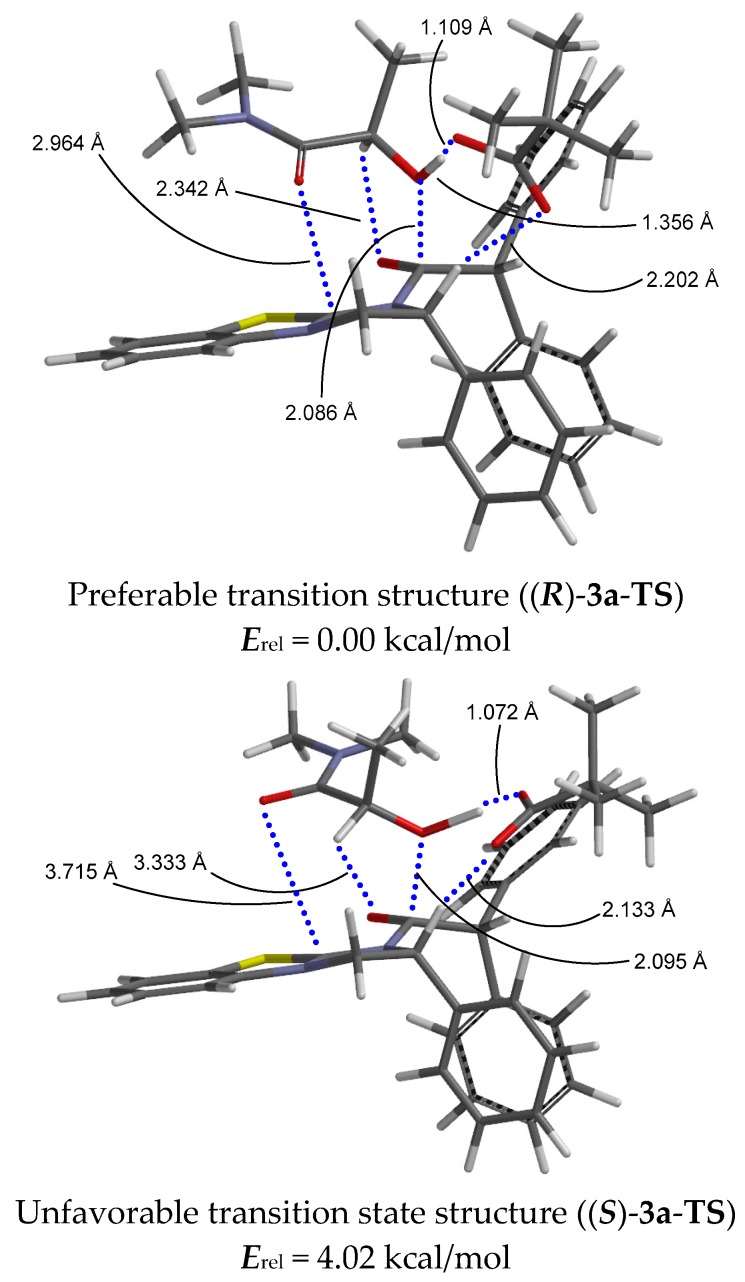
Three-dimensional structures of the calculated transition states ((*R*)-**3a**-**TS** and (*S*)-**3a**-**TS**).

The most stable transition state that affords (*R*)- or (*S*)-2-acyloxy-dimethylamides is shown in Figure 1. It was found that the high selectivity attained in the present KR can be explained by the rapid transformation of (*R*)-**3** into (*R*)-**4** through the stabilized transition state (*R*)-**3**-**TS**, which consists of (*R*)-**3** and the isothiouronium salt derived from the mixed anhydride and (*R*)-BTM. The formation of a C–O bond (between carbonyl carbon of the acid component and oxygen of the hydroxy group) at a distance of 2.086 Å is accompanied by the coordination of oxygen in the carbonyl moiety to hydrogen at the C-2 position of the 2-hydroxydimethylamide at a distance of 2.342 Å, as shown in Figure 1. It was further observed that the length of the cleaved O–H bond (between oxygen and hydrogen in the hydroxyl group) was 1.356 Å. A frequency analysis of (*R*)-**3**-**TS** revealed that the nucleophilic attack of the alcohol to the carbonyl group and the deprotonation of the hydroxyl group with the pivalate anion proceeded via a concerted reaction mechanism because the C–O bond-forming step and the O–H bond-cleaving process occurred simultaneously.

An attractive interaction occurred between oxygen in the amide carbonyl group and the positive electronic charge on the surface of the thiouronium salt, together with coordination of oxygen in the pivalate anion to hydrogen in the hydroxyl group (1.109 Å) and hydrogen at the C-2 position of the dihydroimidazolium salt (2.964 Å). However, complexation of the thiouronium salt with (*R*)-2-hydroxydimethylamide ((*R*)-**3a**), an enantiomer of (*S*)-2-hydroxydimethylamide ((*S*)-**3a**), produced an unstable structure, i.e., (*S*)-**3a**-**TS**; thus, the formation of (*S*)-**3a**-**TS** proceeded slowly due to an energy gap of 4.02 kcal/mol.

**Scheme 3:**
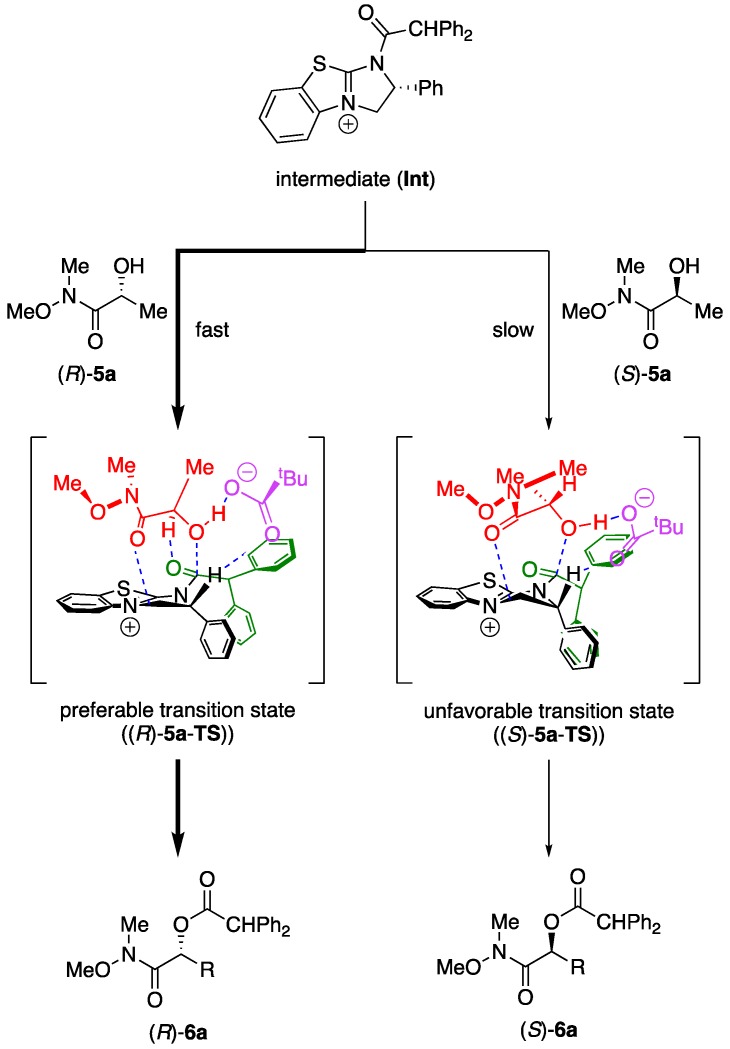
Calculated transition states with a KR of (±)-**5**.

**Figure 2:**
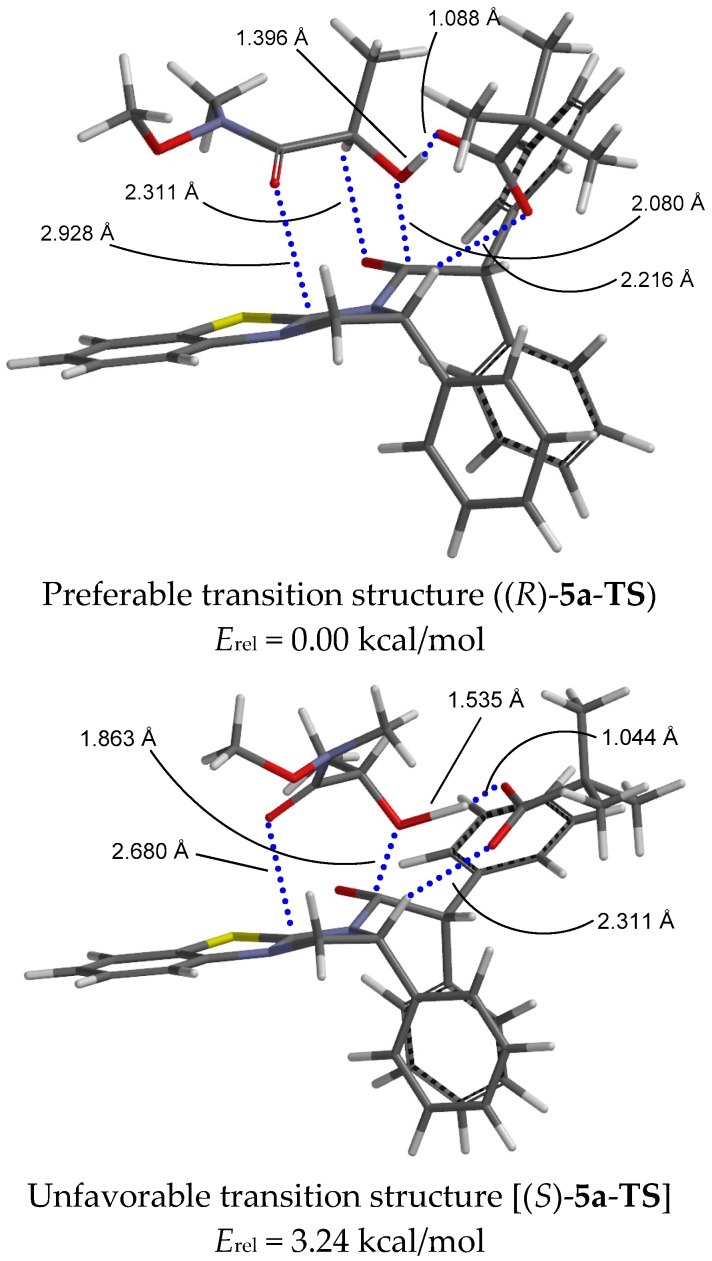
Three-dimensional structures of the calculated transition states ((*R*)-**5a**-**TS** and (*S*)-**5a**-**TS**).

We performed further calculations on the KR of 2-hydroxy-Weinreb amides (Scheme 3). The most stable transition state that affords (*R*)- or (*S*)-2-acyloxy-Weinreb amides is shown in Figure 2 [34]. It was found that the high selectivity attained in the present KR can be explained by the rapid transformation of (*R*)-**5** to (*R*)-**6** through the stabilized transition state (*R*)-**5**-**TS**, which consists of (*R*)-**5** and the isothiouronium salt derived from the mixed anhydride and (*R*)-BTM. The formation of a C–O bond (between carbonyl carbon of the acid component and oxygen of the hydroxy group) at a distance of 2.080 Å is accompanied by the coordination of oxygen in the carbonyl moiety to hydrogen at the C-2 position of the 2-hydroxy-Weinreb amide at a distance of 2.311 Å, as shown in Figure 2. It was further observed that the length of the cleaved O–H bond (between oxygen and hydrogen in the hydroxy group) was 1.396 Å. A frequency analysis of (*R*)-**5**-**TS** revealed that the nucleophilic attack of the alcohol to the carbonyl group and the deprotonation of the hydroxyl group with the pivalate anion proceeded via a concerted reaction mechanism as for the reaction with the 2-hydroxy dimethylamide.

An attractive interaction occurred between oxygen in the amide carbonyl group and the positive electronic charge on the surface of the thiouronium salt, together with coordination of oxygen in the pivalate anion to hydrogen in the hydroxyl group (1.088 Å) and hydrogen at the C-2 position of the dihydroimidazolium salt (2.928 Å). However, complexation of the thiouronium salt with (*R*)-2-hydroxy-Weinreb amide ((*R*)-**5a**), an enantiomer of (*S*)-2-hydroxy-Weinreb amide [(*S*)-**5a**], produced an unstable structure, i.e., (*S*)-**5a**-**TS**; thus, the formation of (*S*)-**5a**-**TS** proceeded slowly due to an energy gap of 3.24 kcal/mol.

**Scheme 4:**
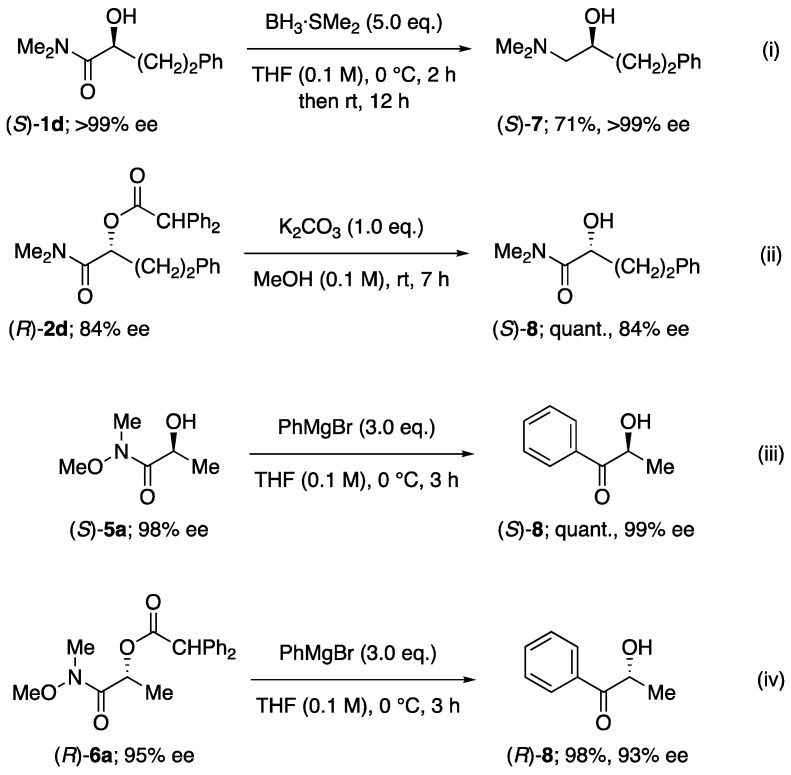
Transformation of chiral 2-hydroxyamides and their esters.

Finally, we attempted to transform the obtained chiral 2-hydroxyamides and their esters based on the KR to demonstrate the synthetic utility of this method (Scheme 4). Reduction of chiral 2-hydroxy-*N*,*N*-dimethylamide (*S*)-**1d** with BH_3_·SMe_2_ in THF afforded amino alcohol (*S*)-**7** in good yield with no loss of chirality (i). When 2-acyloxy-*N*,*N*-dimethylamide (*R*)-**2d** was subjected to solvolysis with K_2_CO_3_ in MeOH, 2-hydroxyamide (*R*)-**1d** was produced in good yield (ii). Treatment of 2-hydroxy-Weinreb amide (*S*)-**5a** and 2-ayloxy-Weinreb amide (*R*)-**6a** with PhMgBr afforded the corresponding 2-hydroxyketone **8** with opposite stereochemistry (iii) and (iv), respectively.

## 3. Materials and Methods

### 3.1. General Information

Optical rotations were determined using a Jasco P-1020 polarimeter. Infrared (IR) spectra were obtained using a Jasco FT/IR-4600 Fourier transform infrared spectrometer. Proton and carbon nuclear magnetic resonance (^1^H and ^13^C NMR) spectra were recorded with chloroform (in CDCl_3_) on the following instruments: JEOL JNM-AL500 (^1^H at 500 MHz and ^13^C at 125 MHz). Mass spectra were determined by a Bruker Daltonics micrOTOF focus (ESI-TOF) mass spectrometer. Thin layer chromatography was performed on Wakogel B5F. HPLC was performed with a Hitachi LaChrom Elite system composed of the Organizer, L-2400 UV Detector, and L-2130 Pump.

### 3.2. Typical Procedure for the Preparation of Optically Active 2-Hydroxy-dimethylamides ***2d*** and ***4a***–***4k***

Condition A; Asymmetric esterification of racemic 2-hydroxy-dimethylamide ((±)-**1d**) with diphenylacetic acid by using Piv_2_O in the presence of (*R*)-BTM was described (Table 1, entry 4): To a solution of racemic 2-hyroxy-dimethylamide ((±)-**1d**) (41.5 mg, 0.20 mmol) in Et_2_O (1.0 mL, 0.20 M) at room temperature were successively added diphenylacetic acid (31.8 mg, 0.15 mmol), Piv_2_O (36.5 μL, 0.18 mmol), *i*-Pr_2_NEt (62.7 μL, 0.36 mmol) and (*R*)-BTM (2.5 mg, 0.01 mmol). The reaction mixture was stirred for 24 h at the same temperature and then it was quenched with saturated aqueous NaHCO_3_. The organic layer was separated, and the aqueous layer was extracted with EtOAc. The combined organic layer was dried over Na_2_SO_4_. After filtration of the mixture and evaporation of the solvent, the crude product was purified by preparative thin layer chromatography on silica to afford the corresponding optically active ester (*R*)-**2d** (38.2 mg, 48% yield, 92% ee) and the recovered optically active alcohol (*S*)-**1d** (19.1 mg, 46% yield, 99% ee) [*s* = 254, Table 1, Entry 4].

Condition B; Asymmetric esterification of racemic 2-hydroxy-dimethylamide ((±)-**1d**) with diphenylacetic anhydride in the presence of (*R*)-BTM was described (Table 1, entry 6): To a solution of racemic 2-hydroxy-dimethylamide ((±)-**1d**) (41.5 mg, 0.20 mmol) in Et_2_O (1.0 mL, 0.2 M) at room temperature were successively added diphenylacetic anhydride (48.8 mg, 0.12 mmol), *i*-Pr_2_NEt (20.9 μL, 0.12 mmol) and (*R*)-BTM (2.5 mg, 0.011 mmol). The reaction mixture was stirred for 24 h at the same temperature and then it was quenched with saturated aqueous NaHCO_3_. The organic layer was separated, and the aqueous layer was extracted with EtOAc. The combined organic layer was dried over Na_2_SO_4_. After filtration of the mixture and evaporation of the solvent, the crude product was purified by preparative thin layer chromatography on silica to afford the corresponding optically active ester (*R*)-**2d** (37.4 mg, 47% yield, 91% ee) and the recovered optically active alcohol (*S*)-**1d** (20.7 mg, 50% yield, 98% ee) [*s* = 95, Table 1, Entry 6].

*(S)-2-Hydroxy-N-methyl-4-phenylbutanamide ((S)-***1a***)*. HPLC (CHIRALPAK IC, *i*-PrOH/hexane = 1/20, flow rate = 1.0 mL/min): *t*R = 47.2 min (57.2%), *t*R = 54.5 min (42.8%); IR (neat): 3309, 1643, 1619, 1550 cm^–1^; ^1^H NMR (CDCl_3_): δ 7.33–7.22 (m, 5H, Ph), 6.85 (brs, 1H, NH), 4.15 (m, 1H, 2-H), 3.92 (d, *J* = 5.5 Hz, 1H, OH), 2.86 (s, 3H, NMe), 2.86–2.79 (m, 2H, 4-H), 2.24–2.12 (m, 1H, 3-H), 2.02–1.95 (m, 1H, 3-H); ^13^C NMR (CDCl_3_): δ 174.8, 141.2, 128.4, 126.0, 71.4, 36.3, 31.2, 25.7; HR MS: calcd for C_12_H_17_NO_2_Na [M + Na]^+^ 216.0995, found 216.1004.

*(S)-N-Benzyl-2-hydroxy-4-phenylbutanamide ((S)-***1b***).* HPLC (CHIRALPAK IC, *i*-PrOH/hexane = 1/9, flow rate = 1.0 mL/min): *t*R = 12.3 min (42.8%), *t*R = 14.6 min (57.2%); IR (KBr): 3366, 3252, 1621, 1538, 1496, 1454, 732, 699 cm^–1^; ^1^H NMR (CDCl_3_): δ 7.37–7.18 (m, 10H, Ph), 7.02 (brs, 1H, NH), 4.46 (dd, *J* = 15.0, 6.0 Hz, 1H, Bn), 4.42 (dd, *J* = 15.0, 6.0 Hz, 1H, Bn), 4.16 (ddd, *J* = 8.0, 5.0, 3.5 Hz, 1H, 2-H), 3.47 (brs, 1H, OH), 2.83–2.73 (m, 2H, 4-H), 2.25–2.15 (m, 1H, 3-H), 2.04–1.94 (m, 1H, 3-H); ^13^C NMR (CDCl_3_): δ 173.8, 141.1, 137.8, 128.7, 128.4, 127.6, 127.5, 126.0, 71.5, 43.1, 36.4, 31.2; HR MS: calcd for C_17_H_19_NO_2_Na [M + Na]^+^ 292.1308, found 292.1312.

*(S)-2-Hydroxy-N,4-diphenylbutanamide ((S)-***1c***).* HPLC (CHIRALPAK IC, *i*-PrOH/hexane = 1/9, flow rate = 1.0 mL/min): *t*R = 8.2 min (86.6%), *t*R = 11.4 min (13.4%); IR (KBr): 3332, 3230, 1656, 1496, 1445, 755, 702 cm^–1^; ^1^H NMR (CDCl_3_): δ 8.42 (s, 1H, NH), 7.57–7.48 (m, 2H, Ph), 7.31–7.09 (m, 8H, Ph), 4.24 (ddd, *J* = 8.3, 4.8, 4.0 Hz, 1H, 2-H), 2.89 (brd, *J* = 4.0 Hz, 1H, OH), 2.82 (d, *J* = 8.0 Hz, 1H, 4-H), 2.81 (d, *J* = 9.5 Hz, 1H, 4-H) 2.32–2.22 (m, 1H, 3-H), 2.11–2.01 (m, 1H, 3-H); ^13^C NMR (CDCl_3_): δ 171.6, 140.9, 137.1, 129.1, 128.6, 128.5, 126.2, 124.6, 119.8, 72.1, 36.2, 31.3; HR MS: calcd for C_16_H_17_NO_2_Na [M + Na]^+^ 278.1151, found 278.1153.

*(S)-2-Hydroxy-N,N-dimethyl-4-phenylbutanamide ((S)-***1d***).* HPLC (CHIRALPAK IC, *i*-PrOH/hexane = 1/4, flow rate = 1.0 mL/min): *t*R = 29.2 min (100.0%); IR (neat): 3457, 1738, 1498, 1456, 1045, 752, 698 cm^–1^; ^1^H NMR (CDCl_3_): δ 7.31–7.16 (m, 5H, Ph), 4.30 (ddd, *J* = 9.0, 7.5, 3.0 Hz, 1H, 2-H), 3.78 (dd, *J* = 7.5, 1.5 Hz, 1H, OH), 2.96 (s, 3H, OMe), 2.87–2.75 (m, 2H, 4-H), 2.80 (s, 3H, NMe), 1.91 (dddd, *J* = 13.5, 9.0, 8.0, 3.0 Hz, 1H, 3-H), 1.78 (dddd, *J* = 13.5, 9.0, 8.5, 5.0 Hz, 1H, 3-H); ^13^C NMR (CDCl_3_): δ 174.2, 141.3, 128.6, 128.4, 126.0, 66.9, 36.4, 36.1, 35.8, 31.2; HR MS: calcd for C_12_H_17_NO_2_Na [M + Na]^+^ 230.1151, found 230.1150.

*(S)-2-Hydroxy-N-methoxy-N-methyl-4-phenylbutanamide ((S)-***1e***) (*=*(S)-***5h***).* HPLC (CHIRALPAK IC, *i*-PrOH/hexane = 1/9, flow rate = 1.0 mL/min): *t*R = 26.7 min (0.8%), *t*R = 29.6 min (99.2%); IR (neat): 3439, 1657, 1487, 1450, 753, 707 cm^–1^; ^1^H NMR (CDCl_3_): δ 7.36–7.19 (m, 5H, Ph), 4.38 (dd, *J* = 7.0, 7.0 Hz, 1H, 2-H), 3.59 (s, 3H, OMe), 3.40 (d, *J* = 7.0 Hz, 1H, OH), 3.24 (s, 3H, NMe), 2.88 (ddd, *J* = 14.0, 9.0, 5.0 Hz, 1H, 4-H), 2.83 (ddd, *J* = 14.0, 8.5, 8.5 Hz, 1H, 4-H), 2.16–2.05 (m, 1H, 3-H), 1.90–1.83 (m, 1H, 3-H); ^13^C NMR (CDCl_3_): δ 175.0, 141.4, 128.6, 128.3, 125.8, 67.7, 61.1, 36.1, 32.4, 31.2; HR MS: calcd for C_12_H_17_NO_3_Na [M + Na]^+^ 246.1101, found 246.1106.

*(R)-2-(Diphenylacetyloxy)-N-methyl-4-phenylbutanamide ((R)-***2a***).* HPLC (CHIRALPAK IC, *i*-PrOH/hexane = 2/3, flow rate = 0.5 mL/min): *t*R = 23.6 min (55.9%), *t*R = 39.3 min (44.1%); IR (neat): 3424, 3309, 1743, 1673, 1542, 748, 709 cm^–1^; ^1^H NMR (CDCl_3_): δ 7.32–7.20 (m, 10H, Ph), 7.17–7.07 (m, 3H, Ph), 6.99–6.97 (m, 2H, Ph), 5.41 (brs, 1H, NH), 5.23 (dd, *J* = 7.0, 4.0 Hz, 1H, 2-H), 4.99 (s, 1H, CHPh_2_), 2.52–2.45 (m, 2H, 3-H), 2.49 (s, 3H, NMe), 2.18–2.00 (m, 1H, 4-H); ^13^C NMR (CDCl_3_): δ 170.7, 169.8, 140.6, 137.8, 137.6, 128.9, 128.7, 128.6, 128.6, 128.4, 128.3, 127.7, 127.6, 126.0, 73.9, 57.1, 33.3, 31.0, 25.7; HR MS: calcd for C_26_H_27_NO_3_Na [M + Na]^+^ 410.1727, found 410.1717.

*(R)-N-Benzyl-2-(diphenylacetyloxy)-4-phenylbutanamide ((R)-***2b***).* HPLC (CHIRALPAK AD-H, *i*-PrOH/hexane = 1/9, flow rate = 1.0 mL/min): *t*R = 23.1 min (58.6%), *t*R = 25.7 min (41.4%); IR (neat): 3308, 1744, 1677, 1496, 1451, 747, 697 cm^−1^; ^1^H NMR (CDCl_3_): δ 7.36–7.18 (m, 16H, Ph), 7.14–7.07 (m, 4H, Ph), 5.88 (t, *J* = 5.5 Hz, 1H, NH), 5.40 (dd, *J* = 7.3, 4.3 Hz, 1H, 2-H), 5.05 (s, 1H, 2′-H), 4.32 (dd, *J* = 14.8, 5.5 Hz, 1H, Bn), 4.24 (dd, *J* = 14.8, 5.5 Hz, 1H, Bn), 2.62 (t, *J* = 8.3 Hz, 2H, 4-H), 2.32–2.17 (m, 2H, 3-H); ^13^C NMR (CDCl_3_): δ 170.7, 169.2, 140.6, 137.7, 137.6, 137.6, 128.9, 128.7, 128.6, 128.6, 128.4, 128.4, 128.3, 127.7, 127.6, 127.6, 127.5, 126.1, 73.9, 57.1, 43.0, 33.4, 31.0; HR MS: calcd for C_31_H_29_NO_3_Na [M + Na]^+^ 486.2040, found 486.2031.

*(R)-2-(Diphenylacetyloxy)-N,4-diphenylbutanamide ((R)-***2c***)*. HPLC (CHIRALPAK IC, *i*-PrOH/hexane = 1/9, flow rate = 1.0 mL/min): *t*R = 11.5 min (10.7%), *t*R = 25.6 min (89.3%); IR (neat): 3312, 1750, 1670, 1494, 1447, 754, 695 cm^–1^; ^1^H NMR (CDCl_3_): δ 7.34–7.00 (m, 21H, Ph, NH), 5.40 (dd, *J* = 6.8, 4.5 Hz, 1H, 2-H), 5.03 (s, 1H, 2′-H), 2.60 (t, *J* = 8.0 Hz, 2H, 4-H), 2.23 (m, 2H, 3-H); ^13^C NMR (CDCl_3_): δ 170.6, 167.3, 140.5, 137.7, 137.7, 136.6, 129.2, 128.8, 128.8, 128.7, 128.5, 128.5, 128.3, 127.8, 127.7, 126.1, 124.7, 119.9, 73.9, 57.2, 33.3, 31.0; HR MS: calcd for C_30_H_27_NO_3_Na [M + Na]^+^ 472.1883, found 472.1874.

*(R)-2-(Diphenylacetyloxy)-N,N-dimethyl-4-phenylbutanamide ((R)-***2d***)*. HPLC (CHIRALPAK IC, *i*-PrOH/hexane = 1/9, flow rate = 1.0 mL/min): *t*R = 17.9 min (4.3%), *t*R = 40.2 min (95.7%); IR (neat): 1737, 1663, 1496, 744, 697 cm^–1^; ^1^H NMR (CDCl_3_): δ 7.38–7.07 (m, 13H, Ph), 6.92–6.85 (m, 2H, Ph), 5.13 (s, 1H, 2′-H), 5.08 (dd, *J* = 10.0, 3.5 Hz, 1H, 2-H), 2.84 (s, 3H, OMe), 2.73 (s, 3H, NMe), 2.59 (ddd, *J* = 14.0, 8.5, 5.0 Hz, 1H, 4-H), 2.41 (ddd, *J* = 14.0, 8.5, 8.5 Hz, 1H, 4-H), 2.11 (dddd, *J* = 14.5, 10.0, 8.5, 5.0 Hz, 1H, 3-H), 1.88 (dddd, *J* = 14.5, 8.5, 8.5, 3.5 Hz, 1H, 3-H); ^13^C NMR (CDCl_3_): δ 172.3, 169.4, 140.3, 138.5, 138.4, 128.8, 128.7, 128.5, 128.4, 128.3, 127.4, 127.2, 126.2, 70.1, 56.7, 36.5, 35.9, 32.4, 31.0; HR MS: calcd for C_26_H_27_NO_3_Na [M + Na]^+^ 424.1883, found 424.1901.

*(R)-2-(Diphenylacetyloxy)-N-methoxy-N-methyl-4-phenylbutanamide ((R)-***2e***) (=(R)-***6h***).* HPLC (CHIRALPAK IC, *i*-PrOH/hexane = 1/9, flow rate = 1.0 mL/min): *t*R = 14.8 min (3.0%), *t*R = 41.3 min (97.0%); IR (neat): 1736, 1674, 1496, 1450, 741, 702 cm^–1^; ^1^H NMR (CDCl_3_): δ 7.51–7.19 (m, *J* = 13H, Ph), 7.05–6.99 (m, 2H, Ph), 5.27 (s, 1H, 2′-H), 5.19 (dd, *J* = 9.5, 3.5 Hz, 1H, 2-H), 3.61 (s, 3H, OMe), 3.21 (s, 3H, NMe), 2.74 (ddd, *J* = 14.0, 8.0, 5.0 Hz, 1H, 4-H), 2.53 (ddd, *J* = 14.0, 8.5, 8.5 Hz, 1H, 4-H), 2.22–2.07 (m, 2H, 3-H); ^13^C NMR (CDCl_3_): δ 172.5, 170.0, 140.3, 138.5, 138.4, 128.8, 128.8, 128.6, 128.4, 128.4, 128.3, 127.3, 127.1, 126.0, 70.8, 61.1, 56.8, 32.1, 31.7, 31.1; HR MS: calcd for C_26_H_27_NO_4_Na [M + Na]^+^ 440.1832, found 440.1852.

*(S)-2-Hydroxy-N,N-dimethylpropanamide ((S)-***3a***).* HPLC (CHIRALPAK IC, *i*-PrOH/hexane = 1/4, flow rate = 1.0 mL/min): *t*R = 17.3 min (5.7%), *t*R = 26.6 min (93.3%); IR (neat): 3417, 1643 cm^−1^; ^1^H NMR (500 MHz, CDCl_3_): δ 4.41 (q, *J* = 6.5 Hz, 1H, 2-H), 3.82 (br s, 1H, OH) 2.95 (s, 3H, NMe), 2.94 (s, 3H, NMe), 1.27 (d, *J* = 6.5 Hz, 3H, 3-H); ^13^C NMR (125 MHz, CDCl_3_): δ 174.9, 64.0, 36.2, 35.8, 20.8; HR MS: calcd for C_5_H_11_NO_2_Na [M + Na]^+^ 140.0682, found 140.0684.

*(S)-2-Hydroxy-N,N-dimethylbutanamide ((S)-***3b***)*. HPLC (CHIRALPAK IC, *i*-PrOH/hexane = 1/4, flow rate = 1.0 mL/min): *t*R = 15.6 min (3.7%), *t*R = 28.2 min (96.3%); IR (neat): 3425, 1642 cm^−1^; ^1^H NMR (500 MHz, CDCl_3_): δ 4.27 (m, 1H, 2-H), 3.68 (d, *J* = 7.5 Hz, 1H, OH), 2.96 (s, 3H, NMe), 2.94 (s, 3H, NMe), 1.67 (m, 1H, 3-H), 1.46 (m, 1H, 3-H), 0.94 (dd, *J* = 7.0, 7.0 Hz, 3H, 4-H); ^13^C NMR (125 MHz, CDCl_3_): δ 174.2, 68.9, 36,3, 35,7, 27,5, 9.1; HR MS: calcd for C_6_H_13_NO_2_Na [M + Na]^+^ 154.0838, found 154.0845.

*(S)-2-Hydroxy-N,N-dimethylpentanamide ((S)-***3c***)*. HPLC (CHIRALPAK IC, *i*-PrOH/hexane = 1/4, flow rate = 1.0 mL/min): *t*R = 17.4 min (82.5%), *t*R = 36.2 min (17.5%); IR (neat): 3425, 1643 cm^−1^; ^1^H NMR (500 MHz, CDCl_3_): δ 4.26 (m, 1H, 2-H), 3.64 (d, *J* = 7.0 Hz, 1H, OH), 2.90 (s, 3H, NMe), 2.89 (s, 3H, NMe), 1.52–1.47 (m, 1H, 3-H), 1.43–1.32 (m, 3H, 3-H, 4-H), 0.84 (dd, *J* = 7.5, 7.5 Hz, 3H, 5-H); ^13^C NMR (125 MHz, CDCl_3_): δ 174.4, 67.6, 36.7, 36.2, 35.7, 18.2, 13.6; HR MS: calcd for C_7_H_15_NO_2_Na [M + Na]^+^ 168.0995, found 168.1000.

*(S)-2-Hydroxy-N,N,3-trimethylbutanamide ((S)-***3d***).* HPLC (CHIRALPAK ID, *i*-PrOH/hexane = 1/4, flow rate = 1.0 mL/min): *t*R = 8.7 min (42.8%), *t*R = 17.4 min (57.2%); IR (neat): 3425, 1643 cm^−1^; ^1^H NMR (500 MHz, CDCl_3_): δ 4.23 (dd, *J* = 7.5, 2.5 Hz, 1H, 2-H), 3.56 (d, *J* = 7.0 Hz, 1H, OH), 2.98 (s, 3H, NMe), 2.97 (s, 3H, NMe), 1.91–1.82 (m, 1H, 3-H), 1.04 (d, *J* = 7.5 Hz, 3H, 4-H), 0.77 (d, *J* = 7.5 Hz, 3H, 4-H); ^13^C NMR (125 MHz, CDCl_3_): δ 173.9, 72.1, 36.5, 35.8, 31.2, 19.7, 15.0; HR MS: calcd for C_7_H_15_NO_2_Na [M + Na]^+^ 168.0995, found 168.0994.

*(S)-2-Hydroxy-N,N-dimethylhexanamide ((S)-***3e***)*. HPLC (CHIRALPAK IC, *i*-PrOH/hexane = 1/4, flow rate = 1.0 mL/min): *t*R = 13.7 min (84.7%), *t*R = 31.2 min (15.3%); IR (neat): 3425, 1643 cm^−1^; ^1^H NMR (500 MHz, CDCl_3_): δ 4.32 (ddd, *J* = 7.5, 7.5, 3.5 Hz, 1H, 2-H), 3.67 (d, *J* = 7.5 Hz, 1H, OH), 2.98 (s, 3H, NMe), 2.96 (s, 3H, NMe), 1.64–1.56 (m, 1H, 3-H), 1.48–1.24 (m, 5H, 3-H, 4-H, 5-H), 0.88 (dd, *J* = 7.5, 7.0 Hz, 3H, 6-H); ^13^C NMR (125 MHz, CDCl_3_): δ 174.5, 67.9, 36.3, 35.8, 34.3, 27.1, 22.4, 13.9; HR MS: calcd for C_8_H_17_NO_2_Na [M + Na]^+^ 182.1151, found 182.1149.

*(S)-2-Hydroxy-N,N,4-trimethylpentanamide ((S)-***3f***).* HPLC (CHIRALPAK IC, *i*-PrOH/hexane = 1/4, flow rate = 1.0 mL/min): *t*R = 14.6 min (89.2%), *t*R = 31.2 min (10.8%); IR (neat): 3425, 1642 cm^−1^; ^1^H NMR (500 MHz, CDCl_3_): δ 4.35 (ddd, *J* = 7.0, 2.5 Hz, 1H, 2-H), 3.59 (d, *J* = 7.0 Hz, 1H, OH), 2.96 (s, 3H, NMe), 2.93 (s, 3H, NMe), 1.94 (ddqq, *J* = 2.5, 4.0, 6.0, 7.0 Hz, 1H, 4-H), 1.38 (ddd, *J* = 14.0, 10.0, 4.0 Hz, 1H, 3-H), 1.27 (ddd, *J* = 14.0, 10.0, 2.5 Hz, 1H, 3-H), 0.95 (d, *J* = 6.0 Hz, 3H, 5-H), 0.91 (d, *J* = 7.0 Hz, 3H, 5-H); ^13^C NMR (125 MHz, CDCl_3_): δ 174.9, 66.4, 43.9, 36.2, 35.8, 24.5, 23.5, 21.2; HR MS: calcd for C_8_H_17_NO_2_Na [M + Na]^+^ 182.1151, found 182.1152.

*(S)-2-Cyclohexyl-2-Hydroxy-N,N-dimethylacetamide ((S)-***3g***).* HPLC (CHIRALPAK IC, *i*-PrOH/hexane = 1/9, flow rate = 1.0 mL/min): *t*R = 11.0 min (53.2%), *t*R = 32.8 min (46.8%); IR (KBr): 3363, 1628 cm^−1^; ^1^H NMR (500 MHz, CDCl_3_): δ 4.19 (d, *J* = 1.5 Hz, 1H, 2-H), 3.54 (br s, 1H, OH), 2.98 (s, 3H, NMe), 2.97 (s, 3H, NMe), 1.77–1.72 (m, 2H, *c*-Hex), 1.62–1.60 (m, 2H, *c*-Hex), 1.50–1.37 (m, 3H, *c*-Hex), 1.26–1.05 (m, 4H, *c*-Hex); ^13^C NMR (125 MHz, CDCl_3_): δ 173.8, 72.0, 41.4, 36.6, 35.8, 29.8, 26.4, 26.0, 25.9, 25.5; HR MS: calcd for C_10_H_19_NO_2_Na [M + Na]^+^ 208.1308, found 208.1311.

*(S)-3-(tert-Butyldimethylsiloxy)-2-hydroxy-N,N-dimethylpropanamide ((S)-***3i***).* HPLC (CHIRALPAK IC, *i*-PrOH/hexane = 1/9, flow rate = 1.0 mL/min): *t*R = 9.0 min (70.6%), *t*R = 13.3 min (29.4%); IR (neat): 3278, 1635 cm^−1^; ^1^H NMR (500 MHz, CDCl_3_): δ 4.49 (ddd, *J* = 9.5, 6.0, 2.0 Hz, 1H, 2-H), 3.78 (dd, *J* = 10.0, 5.0 Hz, 1H, 3-H), 3.65 (d, *J* = 7.5 Hz, 1H, OH), 3.63 (dd, *J* = 10.0, 7.5 Hz, 1H, 3-H), 3.05 (s, 3H, NMe), 2.99 (s, 3H, NMe), 0.86 (s, 9H, TBS), 0.04 (s, 3H, TBS), 0.03 (s, 3H, TBS); ^13^C NMR (125 MHz, CDCl_3_): δ 172.8, 68.7, 66.3, 36.8, 35.9, 25.8, 18.3, –5.5; HR MS: calcd for C_11_H_25_NO_3_SiNa [M + Na]^+^ 270.1496, found 270.1509.

*(S)-4-(tert-Butyldimethylsiloxy)-2-hydroxy-N,N-dimethylbutanamide ((S)-***3j***).* HPLC (CHIRALPAK IC, *i*-PrOH/hexane = 1/4, flow rate = 1.0 mL/min): *t*R = 11.4 min (3.5%), *t*R = 25.0 min (96.5%); IR (neat): 3363, 1643 cm^−1^; ^1^H NMR (500 MHz, CDCl_3_): δ 4.55–4.51 (m, 1H, 2-H), 3.85 (ddd, *J* = 10.0, 10.0, 3.5 Hz, 1H, 4-H), 3.75 (ddd, *J* = 10.0, 10.0, 3.5 Hz, 1H, 4-H), 3.65 (d, *J* = 7.5 Hz, 1H, OH), 2.98 (s, 6H, NMe), 1.85–1.80 (m, 1H, 3-H), 1.58–1.51 (m, 1H, 3-H), 0.88 (s, 9H, TBS), 0.06 (s, 3H, TBS), 0.05 (s, 3H, TBS); ^13^C NMR (125 MHz, CDCl_3_): δ 174.6, 64.9, 59.2, 38.3, 36.1, 35.8, 25.8, 18.2, –5.5; HR MS: calcd for C_12_H_27_NO_3_SiNa [M + Na]^+^ 284.1652, found 284.1645.

*(S)-5-(tert-Butyldimethylsiloxy)-2-hydroxy-N,N-dimethylpentanamide ((S)-***3k***).* HPLC (CHIRALPAK IC, *i*-PrOH/hexane = 1/9, flow rate = 1.0 mL/min): *t*_R_ = 12.2 min (97.2%), *t*_R_ = 30.3 min (2.8%); IR (neat): 3425, 1643 cm^−1^; ^1^H NMR (500 MHz, CDCl_3_): δ 4.36 (m, 1H, 2-H), 3.71 (d, *J* = 7.0 Hz, 1H, OH), 3.69–3.59 (m, 2H, 5-H), 2.98 (s, 3H, NMe), 2.96 (s, 3H. NMe), 1.80–1.73 (m, 1H, 3-H), 1.68–1.62 (m, 2H, 4-H), 1.52–1.44 (m, 1H, 3-H), 0.86 (s, 9H, TBS), 0.01 (s, 6H, TBS); ^13^C NMR (125 MHz, CDCl_3_): δ 174.4, 67.6, 62.3, 36.3, 35.8, 30.9, 28.0, 25.8, 18.2, –5.4; HR MS: calcd for C_13_H_29_NO_3_SiNa [M + Na]^+^ 298.1809 found 298.1805.

*(R)-2-(Diphenylaceloxy)-N,N-dimethylpropanamide ((R)-***4a***).* HPLC (CHIRALPAK IC, *i*-PrOH/hexane = 20/80, flow rate = 1.0 mL/min): *t*_R_ = 17.3 min (5.7%), *t*_R_ = 24.0 min (94.3%); IR (neat): 1736, 1666, 1496, 1458, 741, 702 cm^−1^; ^1^H NMR (500 MHz, CDCl_3_): δ 7.35–7.21 (m, 10H, Ph), 5.43 (q, *J* = 6.0 Hz, 1H, 2-H), 5.12 (s, 1H, 2′-H), 2.93 (s, 6H, NMe_2_), 1.41 (d, *J* = 6.0 Hz, 3H, 3-H); ^13^C NMR (125 MHz, CDCl_3_): δ 172.1, 169.7, 138.4, 138.3, 128.7, 128.6, 128.5, 128.4, 127.2, 127.1, 67.7, 56.6, 36.6, 35.6, 16.5; HR MS: calcd for C_19_H_21_NO_3_Na [M + Na]^+^ 334.1414, found 334.1407.

*(R)-2-(Diphenylacetyloxy)-N,N-dimethylbutanamide ((R)-***4b***).* HPLC (CHIRALPAK IC, *i*-PrOH/hexane = 20/80, flow rate = 1.0 mL/min): *t*_R_ = 15.6 min (3.7%), *t*_R_ = 28.2 min (96.3%); IR (neat): 1736, 1658, 1496, 1458, 741, 702 cm^−1^; ^1^H NMR (500 MHz, CDCl_3_): δ 7.34–7.18 (m, 10H, Ph), 5.21 (dd, *J* = 7.5, 5.5 Hz, 1H, 2-H), 5.11 (s, 1H, 2′-H), 2.96 (s, 3H, NMe), 2.91 (s, 3H, NMe) 1.79–1.71 (m, 2H, 3-H), 0.84 (t, *J* = 7.5 Hz, 3H, 4-H); ^13^C NMR (125 MHz, CDCl_3_): δ 172.4, 169.3, 138.5, 128.7, 128.6, 128.5, 128.4, 127.2, 127.1, 72.5, 56.7, 36.7, 35.8, 24.3, 9.6; HR MS: calcd for C_20_H_23_NO_3_Na [M + Na]^+^ 348.1570, found 348.1577.

*(R)-2-(Diphenylacetyloxy)-N,N-dimethylpentanamide ((R)-***4c***).* HPLC (CHIRALPAK IC, *i*-PrOH/hexane = 20/80, flow rate = 1.0 mL/min): *t*_R_ = 13.8 min (2.9%), *t*_R_ = 27.6 min (97.1%); IR (neat): 1736, 1666, 1496, 1458, 741, 702 cm^−1^; ^1^H NMR (500 MHz, CDCl_3_): δ 7.36–7.21 (m, 10H, Ph), 5.31 (dd, *J* = 8.5, 4.5 Hz, 1H, 2-H), 5.14 (s, 1H, 2′-H), 3.00 (s, 3H, NMe), 2.94 (s, 3H, NMe), 1.82–1.76 (m, 1H, 3-H), 1.70–1.64 (m, 1H, 3-H), 1.38–1.22 (m, 2H, 4-H), 0.85 (t, *J* = 7.5 Hz, 3H, 5-H); ^13^C NMR (125 MHz, CDCl_3_): δ 172.4, 169.5, 138.5, 138.5, 128.7, 128.6, 128.5, 128.4, 127.2, 127.1, 71.0, 56.6, 36.7, 35.9, 32.9, 18.4, 13.5; HR MS: calcd for C_21_H_25_NO_3_Na [M + Na]^+^ 362.1727, found 362.1733.

*(R)-2-(Diphenylacetyloxy)-N,N,3-trimethylbutanamide ((R)-***4d***).* HPLC (CHIRALPAK ID, *i*-PrOH/hexane = 20/80, flow rate = 1.0 mL/min): *t*_R_ = 8.7 min (90.9%), *t*_R_ = 18.4 min (9.1%); IR (neat): 1736, 1658, 1496, 1458, 748, 702 cm^−1^; ^1^H NMR (500 MHz, CDCl_3_): δ 7.31–7.14 (m, 10H, Ph), 5.08 (s, 1H, 2′-H), 4.99 (d, *J* = 7.5 Hz, 1H, 2-H), 3.00 (s, 3H, NMe), 2.89 (s, 3H, NMe), 2.08 (m, 1H, 3-H), 0.80 (d, *J* = 7.5 Hz, 3H, 4-H), 0.78 (d, *J* = 6.0 Hz, 3H, 4-H); ^13^C NMR (125 MHz, CDCl_3_): δ 172.5, 169.1, 138.5, 138.5, 128.7, 128.7, 128.5, 128.3, 127.2, 127.1, 75.6, 56.8, 37.0, 35.9, 30.1, 18.4, 17.7; HR MS: calcd for C_21_H_25_NO_3_Na [M + Na]^+^ 362.1727, found 362.1710.

*(R)-2-(Diphenylacetyloxy)-N,N-dimethylhexanamide ((R)-***4e***).* HPLC (CHIRALPAK IC, *i*-PrOH/hexane = 20/80, flow rate = 1.0 mL/min): *t*_R_ = 13.4 min (2.7%), *t*_R_ = 30.0 min (97.3%); IR (neat): 1736, 1666, 1496, 1458, 741, 702 cm^−1^; ^1^H NMR (500 MHz, CDCl_3_): δ 7.37–7.21 (m, 10H, Ph), 5.30 (dd, *J* = 5.0, 5.0 Hz, 1H, 2-H), 5.14 (s, 1H, 2′-H), 3.00 (s, 3H, NMe), 2.94 (s, 3H, NMe), 1.80 (m, 1H, 3-H), 1.70 (m, 1H, 3-H), 1.27–1.20 (m, 4H, 4-H, 5-H), 0.77 (t, *J* = 6.5, 6.0 Hz, 3H, 3-H); ^13^C NMR (125 MHz, CDCl_3_): δ 172.4, 169.6, 138.5, 138.5, 128.7, 128.7, 128.6, 128.4, 127.2, 127.1, 71.2, 56.7, 36.7, 35.9, 30.6, 27.2, 22.1, 13.7; HR MS: calcd for C_22_H_27_NO_3_Na [M + Na]^+^ 376.1883, found 376.1898.

*(R)-2-(Diphenylacetyloxy)-N,N,4-trimethylpentanamide ((R)-***4f***).* HPLC (CHIRALPAK IC, *i*-PrOH/hexane = 20/80, flow rate = 1.0 mL/min): *t*_R_ = 14.8 min (2.5%), *t*_R_ = 31.3 min (97.5%); IR (neat): 1736, 1666, 1496, 1458, 741, 702 cm^−1^; ^1^H NMR (500 MHz, CDCl_3_): δ 7.33–7.18 (m, 10H, Ph), 5.32 (dd, *J* = 10.4, 3.4 Hz, 1H, 2-H), 5.12 (s, 1H, 2′-H), 2.98 (s, 3H, NMe), 2.91 (s, 3H, NMe), 1.79 (ddd, *J* = 14.6, 10.4, 4.6, 1H, 3-H), 1.54–1.51 (m, 1H, 4-H), 1.38 (ddd, *J* = 14.0, 9.2, 3.4 Hz, 1H, 3-H), 0.81 (d, *J* = 6.7 Hz, 3H, 5-H), 0.79 (d, *J* = 6.4 Hz, 3H, 5-H); ^13^C NMR (125 MHz, CDCl_3_): δ 172.5, 169.8, 138.5, 138.4, 128.7, 128.7, 128.5, 128.4, 127.2, 127.1, 69.9, 56.7, 39.6, 36.6, 35.9, 24.4, 23.0, 21.3; HR MS: calcd for C_22_H_27_NO_3_Na [M + Na]^+^ 376.1883, found 376.1873.

*(R)-2-Cyclohexyl-2-(diphenylacetyloxy)-N,N-dimethylacetamide ((R)-***4g***).* HPLC (CHIRALPAK IC, *i*-PrOH/hexane = 40/60, flow rate = 0.75 mL/min): *t*_R_ = 11.2 min (16.8%), *t*_R_ = 31.3 min (83.2%); IR (neat): 1736, 1658, 1496, 1450, 748, 702 cm^−1^; ^1^H NMR (500 MHz, CDCl_3_): δ 7.29–7.13 (m, 10H, Ph), 5.06 (s, 1H, 2′-H), 5.00 (d, *J* = 7.3 Hz, 1H, 2-H), 3.00 (s, 3H, NMe), 2.88 (s, 3H, NMe), 1.77 (m, 1H, 3-H), 1.58–1.45 (m, 5H, *c*-Hex), 1.17–0.80 (m, 5H, *c*-Hex); ^13^C NMR (125 MHz, CDCl_3_): δ 172.5, 169.1, 138.6, 138.5, 128.8, 128.8, 128.6, 128.4, 127.2, 127.1, 75.0, 56.7, 39.5, 37.1, 35.9, 28.5, 28.2, 26.0, 25.8, 25.5; HR MS: calcd for C_24_H_29_NO_3_Na [M + Na]^+^ 402.2040, found 402.2047.

*(R)-3-(tert-Butyldimethylsiloxy)-2-(diphenylacetyloxy)-N,N-dimethylpropanamide ((R)-***4i***).* HPLC (CHIRALPAK IC, *i*-PrOH/hexane = 20/80, flow rate = 1.0 mL/min): *t*_R_ = 11.4 min (9.0%), *t*_R_ = 13.3 min (91.0%); IR (neat): 1743, 1658, 1496, 1458, 741, 702 cm^−1^; ^1^H NMR (500 MHz, CDCl_3_): δ 7.36–7.23 (m, 10H, Ph), 5.49 (t, *J* = 6.0 Hz, 1H, 2-H), 5.15 (s, 1H, 2′-H), 3.90 (m, 2H, 3-H), 3.10 (s, 3H, NMe), 2.97 (s, 3H, NMe), 0.85 (s, 9H, TBS), 0.02 (s, 3H, TBS), 0.00 (s, 3H, TBS); ^13^C NMR (125 MHz, CDCl_3_): δ 172.2, 168.1, 138.4, 138.4, 128.7, 128.6, 128.5, 127.3, 127.2, 71.6, 62.9, 56.6, 37.0, 36.0, 25.7, 18.1, 5.6, 5.7; HR MS: calcd for C_25_H_35_NO_4_SiNa [M + Na]^+^ 464.2228, found 464.2222.

*(R)-4-(tert-Butyldimethylsiloxy)-2-(diphenylacetyloxy)-N,N-dimethylbutanamide ((R)-***4j***).* HPLC (CHIRALPAK IC, *i*-PrOH/hexane = 20/80, flow rate = 1.0 mL/min): *t*_R_ = 11.4 min (3.5%), *t*_R_ = 25.0 min (96.5%); IR (neat): 1743, 1666, 1496, 1466, 748, 717 cm^−1^; ^1^H NMR (500 MHz, CDCl_3_): δ 7.38–7.22 (m, 10H, Ph), 5.54 (dd, *J* = 9.5, 3.0 Hz, 1H, 2-H), 5.17 (s, 1H, 2′-H), 3.60 (dt, *J* = 10.0, 5.0 Hz, 1H, 4-H), 3.48 (dt, *J* = 10.0, 3.5 Hz, 1H, 4-H), 3.08 (s, 3H, NMe), 2.98 (s, 3H, NMe), 1.98 (m, 1H, 3-H), 1.89 (m, 1H, 3-H) 0.86 (s, 9H, TBS), 0.02 (s, 3H, TBS), 0.06 (s, 3H, TBS); ^13^C NMR (125 MHz, CDCl_3_): δ 172.3, 169.8, 138.6, 138.5, 128.8, 128.7, 128.6, 128.4, 127.2, 127.1, 68.0, 58.4, 56.7, 36.6, 35.8, 34.2, 25.8, 18.1, 5.6, 5.7; HR MS: calcd for C_26_H_37_NO_4_SiNa [M + Na]^+^ 478.2384, found 478.2386.

*(R)-5-(tert-Butyldimethylsiloxy)-2-(Diphenylacetyloxy)-N,N-dimethylpentanamide ((R)-***4k***).* HPLC (CHIRALPAK IC, *i*-PrOH/hexane = 20/80, flow rate = 1.0 mL/min): *t*_R_ = 12.2 min (2.4%), *t*_R_ = 29.7 min (97.6%); IR (neat): 1751, 1666, 1496, 1458, 748, 702 cm^−1^; ^1^H NMR (500 MHz, CDCl_3_): δ 7.37–7.22 (m, 10H, Ph), 5.35 (t, *J* = 6.5 Hz, 1H, 2-H), 5.15 (s, 1H, 2′-H), 3.54 (t, *J* = 6.0 Hz, 2H, 5-H), 3.02 (s, 3H, NMe), 2.95 (s, 3H, NMe), 1.84 (dt, *J* = 6.5, 6.5 Hz, 2H, 3-H), 1.55–1.39 (m, 2H, 4-H), 0.87 (s, 9H, TBS), 0.01 (s, 6H, TBS); ^13^C NMR (125 MHz, CDCl_3_): δ 172.4, 169.5, 138.5, 138.5, 128.8, 128.7, 128.6, 128.4, 127.2, 127.1, 71.1, 62.0, 56.7, 36.7, 35.9, 28.0, 27.3, 25.9, 18.2, 5.4; HR MS: calcd for C_27_H_39_NO_4_SiNa [M + Na]^+^ 492.2541, found 492.2554.

*(S)-2-Hydroxy-N-methoxy-N-methylpropanamide ((S)-***5a***).* HPLC (CHIRALPAK IC, *i*-PrOH/hexane = 1/9, flow rate = 0.5 mL/min): *t*_R_ = 16.6 min (99.2%), *t*_R_ = 27.3 min (0.8%); IR (neat): 3443, 1662 cm^–1^; ^1^H NMR (CDCl_3_): δ 4.42 (dq, *J* = 7.0, 7.0 Hz, 1H, 2-H), 3.65 (s, 3H, OMe), 3.42 (d, *J* = 7.0 Hz, 1H, OH), 2.81 (s, 3H, NMe), 1.29 (d, *J* = 7.0 Hz, 3H, 3-H); ^13^C NMR (CDCl_3_): δ 175.6, 64.8, 61.1, 32.2, 20.8; HR MS: calcd for C_5_H_11_NO_3_Na [M + Na]^+^ 156.0631, found 156.0634.

*(S)-2-Hydroxy-N-methoxy-N-methylbutanamide ((S)-***5b***).* HPLC (CHIRALPAK IC, *i*-PrOH/hexane = 1/9, flow rate = 1.0 mL/min): *t*_R_ = 13.6 min (92.4%), *t*_R_ = 41.3 min (7.6%); IR (neat): 3448, 1658 cm^–1^; ^1^H NMR (CDCl_3_): δ 4.33 (ddd, *J* = 7.5, 7.5, 3.5 Hz, 1H, 2-H), 3.69 (s, 3H, OMe), 3.24 (d, *J* = 7.5 Hz, 1H, OH), 3.22 (s, 3H, NMe), 1.76 (dqd, *J* = 14.5, 7.5, 3.5 Hz, 1H, 3-H), 1.55 (ddq, *J* = 14.5, 7.5, 7.5 Hz, 1H, 3-H), 0.95 (dd, *J* = 7.5, 7.5 Hz, 3H, 4-H); ^13^C NMR (CDCl_3_): δ 175.0, 69.6, 61.2, 32.3, 27.6, 9.1; HR MS: calcd for C_6_H_13_NO_3_Na [M + Na]^+^ 170.0788, found 170.0793.

*(S)-2-Hydroxy-N-methoxy-N-methylpentanamide ((S)-***5c***).* HPLC (CHIRALPAK IC, *i*-PrOH/hexane = 1/9, flow rate = 1.0 mL/min): *t*_R_ = 13.1 min (96.8%), *t*_R_ = 33.4 min (3.2%); IR (neat): 3464, 1658 cm^–1^; ^1^H NMR (CDCl_3_): δ 4.43–4.24 (m, 1H, 2-H), 3.66 (dd, *J* = 14.0, 14.0 Hz, 3H, OMe), 3.28–3.20 (m, 1H, OH), 3.19 (dd, *J* = 14.0, 14.0 Hz, 3H, NMe), 1.71–1.57 (m, 1H, 3-H), 1.53–1.35 (m, 3H, 3-H, 4-H), 0.89 (dddd, *J* = 15.0, 15.0, 7.5, 7.5 Hz, 3H, 5-H); ^13^C NMR (CDCl_3_): δ 175.2, 68.3, 61.1, 36.8, 36.7, 32.3, 18.2, 18.1, 13.6; HR MS: calcd for C_7_H_15_NO_3_Na [M + Na]^+^ 184.0944, found 184.0941.

*(S)-2-Hydroxy-N-methoxy-N,3-dimethylbutanamide ((S)-***5d***).* HPLC (CHIRALPAK IC, *i*-PrOH/hexane = 1/9, flow rate = 1.0 mL/min): *t*_R_ = 11.1 min (53.4%), *t*_R_ = 31.8 min (46.6%); IR (neat): 3455, 1656 cm^–1^; ^1^H NMR (CDCl_3_): δ 4.23 (dd, *J* = 8.0, 2.5 Hz, 1H, 2-H), 3.67 (s, 3H, OMe), 3.21 (s, 3H, NMe), 3.13 (d, *J* = 8.0 Hz, 1H, OH), 2.05–1.93 (m, 1H, 3-H), 1.00 (d, *J* = 7.0 Hz, 3H, 4-H), 0.78 (d, *J* = 7.0 Hz, 3H, 4-H); ^13^C NMR (CDCl_3_): δ 174.6, 72.8, 32.3, 31.3, 19.6, 15.2; HR MS: calcd for C_7_H_15_NO_3_Na [M + Na]^+^ 184.0944, found 184.0949.

*(S)-2-Hydroxy-N-methoxy-N-methylhexanamide ((S)-***5e***).* HPLC (CHIRALPAK IC, *i*-PrOH/hexane = 1/9, flow rate = 1.0 mL/min): *t*_R_ = 10.3 min (84.3%), *t*_R_ = 26.1 min (15.7%); IR (neat): 3449, 1658 cm^–1^; ^1^H NMR (CDCl_3_): δ 4.37–4.34 (m, 1H, 2-H), 3.68 (s, 3H, OMe), 3.23 (s, 1H, OH), 3.21 (s, 3H, NMe), 1.75–1.64 (m, 1H, 3-H), 1.55–1.21 (m, 3H, 3-H, 4-H), 0.88 (dd, *J* = 7.5, 7.5 Hz, 3H, 5-H); ^13^C NMR (CDCl_3_): δ 175.3, 68.6, 61.2, 34.3, 32.3, 27.0, 22.3, 13.8; HR MS: calcd for C_8_H_17_NO_3_Na [M + Na]^+^ 198.1101, found 198.1110.

*(S)-2-Hydroxy-N-methoxy-N,4-dimethylpentanamide ((S)-***5f***).* HPLC (CHIRALPAK IC, *i*-PrOH/hexane = 1/9, flow rate = 0.5 mL/min): *t*_R_ = 20.0 min (82.3%), *t*_R_ = 50.9 min (4.8%); IR (neat): 3447, 1660 cm^–1^; ^1^H NMR (CDCl_3_): δ 4.39 (dd, *J* = 8.0, 8.0 Hz, 1H, 2-H), 3.68 (s, 3H, OMe), 3.20 (s, 3H, NMe), 3.15 (d, *J* = 8.0 Hz, 1H, OH), 1.95–1.84 (m, 1H, 4-H), 1.48–1.33 (m, 2H, 3-H), 0.93 (d, *J* = 7.0 Hz, 3H, 5-H), 0.91 (d, *J* = 6.5 Hz, 3H, 5-H); ^13^C NMR (CDCl_3_): δ 175.7, 67.2, 61.1, 43.9, 32.4, 24.5, 23.5, 21.2; HR MS: calcd for C_8_H_17_NO_3_Na [M + Na]^+^ 198.1101, found 198.1097.

*(S)-2-Cyclohexyl-2-hydroxy-N-methoxy-N-methylacetamide ((S)-***5h***).* HPLC (CHIRALPAK IC, *i*-PrOH/hexane = 1/9, flow rate = 1.0 mL/min): *t*_R_ = 10.2 min (52.5%), *t*_R_ = 40.4 min (47.5%); IR (neat): 3451, 1656 cm^–1^; ^1^H NMR (CDCl_3_): δ 4.20 (d, *J* = 4.0 Hz, 1H, 2-H), 3.67 (s, 3H, OMe), 3.21 (s, 3H, NMe), 3.13 (d, *J* = 8.0 Hz, 1H, OH), 1.76–1.53 (m, 5H, *c*-Hex), 1.47–1.30 (m, 2H, *c*-Hex), 1.26–1.03 (m, 4H, *c*-Hex); ^13^C NMR (CDCl_3_): δ 174.4, 72.6, 61.1, 41.4, 32.2, 29.6, 26.3, 26.0, 25.9; HR MS: calcd for C_10_H_19_NO_3_Na [M + Na]^+^ 224.1257, found 224.1248.

*(S)-3-(tert-Butyldimethylsiloxy)-2-hydroxy-N-methoxy-N-methylpropanamide ((S)-***5i***).* HPLC (CHIRALPAK IC, *i*-PrOH/hexane = 1/9, flow rate = 0.5 mL/min): *t*_R_ = 13.7 min (75.4%), *t*_R_ = 20.2 min (24.6%); IR (neat): 3447, 1665 cm^–1^; ^1^H NMR (CDCl_3_): δ 4.52–4.35 (m, 1H, 2-H), 3.86 (dd, *J* = 10.0, 3.5 Hz, 1H, 3-H), 3.81 (dd, *J* = 10.0, 3.5 Hz, 1H, 3-H), 3.70 (dd, *J* = 15.0, 15.0 Hz, 3H, OMe), 3.48 (ddd, *J* = 15.0, 15.0, 8.5 Hz, 1H, OH), 3.23 (dd, *J* = 15.0, 15.0 Hz, 3H, NMe), 0.86 (dd, *J* = 15.0, 15.0 Hz, 9H, TBS), 0.04 (dd, *J* = 15.0, 15.0 Hz, 3H, TBS), 0.03 (s, 3H, TBS); ^13^C NMR (CDCl_3_): δ 172.3, 70.2, 65.2, 61.2, 32.4, 25.8, 18.3, −5.4, −5.5; HR MS: calcd for C_11_H_25_NO_4_SiNa [M + Na]^+^ 286.1445, found 286.1431.

*(S)-4-(tert-Butyldimethylsiloxy)-2-hydroxy-N-methoxy-N-methylbutanamide ((S)-***5j***).* HPLC (CHIRALPAK IC, *i*-PrOH/hexane = 1/9, flow rate = 1.0 mL/min): *t*_R_ = 9.6 min (95.2%), *t*_R_ = 23.1 min (4.8%); IR (neat): 3451, 1662, cm^–1^; ^1^H NMR (CDCl_3_): δ 4.62–4.48 (m, 1H, 2-H), 3.90–3.74 (m, 2H, 4-H), 3.70 (dd, *J* = 15.0, 15.0 Hz, 3H, OMe), 3.28 (d, *J* = 7.0 Hz, 1H, OH), 3.23 (ddd, *J* = 14.5, 14.5, 5.0 Hz, 3H, NMe), 2.05–1.88 (m, 1H, 3-H), 1.68–1.55 (m, 1H, 3-H), 0.89 (dd, *J* = 15.0, 15.0 Hz, 9H, TBS), 0.06 (ddd, *J* = 14.5, 14.5, 5.0 Hz, 3H, TBS), 0.05 (s, 3H, TBS); ^13^C NMR (CDCl_3_): δ 175.3, 65.9, 61.3, 59.2, 37.6, 32.5, 25.9, 18.2, −5.4, −5.5; HR MS: calcd for C_12_H_27_NO_4_SiNa [M + Na]^+^ 300.1602, found 300.1607.

*(S)-4-(tert-Butyldimethylsiloxy)-2-hydroxy-N-methoxy-N-methylpentanamide ((S)-***5k***).* HPLC (CHIRALPAK IC, *i*-PrOH/hexane = 1/9, flow rate = 1.0 mL/min): *t*_R_ = 8.7 min (99.6%), *t*_R_ = 21.7 min (0.4%); IR (neat): 3464, 1658 cm^–1^; ^1^H NMR (CDCl_3_): δ 4.47–4.33 (m, 1H, 2-H), 3.70 (s, 3H, OMe), 3.64 (td, *J* = 6.0, 2.5 Hz, 1H, 5-H), 3.30 (d, *J* = 8.0 Hz, 1H, OH), 3.23 (s, 3H, NMe), 1.87–1.78 (m, 1H, 3-H), 1.70–1.51 (m, 3H, 3-H, 4-H), 0.87 (s, 9H, TBS), 0.03 (s, 6H, TBS); ^13^C NMR (CDCl_3_): δ 175.1, 68.5, 62.7, 61.2, 32.4, 31.2, 28.3, 25.9, 18.3, –5.3; HR MS: calcd for C_13_H_29_NO_4_SiNa [M + Na]^+^ 314.1758, found 314.1748.

*(R)-2-(Diphenylacetyloxy)-N-methoxy-N-methylpropanamide ((R)-***6a***).* HPLC (CHIRALPAK IC, *i*-PrOH/hexane = 1/4, flow rate = 1.0 mL/min): *t*_R_ = 18.9 min (26.5%), *t*_R_ = 26.5 in (96.4%); IR (neat): 1736, 1673, 1489, 1458, 741, 702 cm^–1^; ^1^H NMR (CDCl_3_): δ 7.36–7.19 (m, 10H, Ph), 5.39 (q, *J* = 6.8 Hz, 1H, 2-H), 5.13 (s, 1H, 2′-H), 3.73 (s, 3H, OMe), 3.18 (s, 3H, NMe), 1.41 (d, *J* = 6.8 Hz, 3H, 3-H); ^13^C NMR (CDCl_3_): δ 172.5, 170.6, 138.6, 138.5, 128.8, 128.7, 128.6, 128.4, 127.2, 127.1, 68.3, 56.6, 32.1, 16.3; HR MS: calcd for C_19_H_21_NO_4_Na [M + Na]^+^ 350.1363, found 350.1350.

*(R)-2-(Diphenylacetyloxy)-N-methoxy-N-methylbutanamide ((R)-***6b***).* HPLC (CHIRALPAK IC, *i*-PrOH/hexane = 1/4, flow rate = 1.0 mL/min): *t*_R_ = 13.6 min (2.2%), *t*_R_ = 30.8 min (97.8%); IR (neat): 1736, 1676, 1486, 1454, 749, 699 cm^–1^; ^1^H NMR (CDCl_3_): δ 7.39–7.19 (m, 10H, Ph), 5.25 (t, *J* = 7.0 Hz, 1H, 2-H), 5.16 (s, 1H, 2′-H), 3.76 (s, 3H, OMe), 3.20 (s, 3H, NMe), 1.85–1.76 (m, 2H, 3-H), 0.88 (t, *J* = 7.0 Hz, 3H, 4-H); ^13^C NMR (CDCl_3_): δ 172.7, 170.0, 138.6, 138.5, 128.8, 128.8, 128.6, 128.4, 127.2, 127.1, 73.0, 61.2, 56.7, 32.0, 24.1, 9.7; HR MS: calcd for C_20_H_23_NO_4_Na [M + Na]^+^ 364.1519, found 364.1537.

*(R)-2-(Diphenylacetyloxy)-N-methoxy-N-methylpentanamide ((R)-***6c***).* HPLC (CHIRALPAK IC, *i*-PrOH/hexane = 1/9, flow rate = 1.0 mL/min): *t*_R_ = 13.2 min (1.9%), *t*_R_ = 32.7 min (98.1%); IR (neat): 1736, 1678, 1602, 1497, 1459, 740, 698 cm^–1^; ^1^H NMR (CDCl_3_): δ 7.35–7.16 (m, 10H, Ph), 5.28 (dd, *J* = 9.0, 3.5 Hz, 1H, 2-H), 5.12 (s, 1H, 2′-H), 3.73 (s, 3H, OMe), 3.16 (s, 3H, NMe), 1.80–1.61 (m, 2H, 3-H), 1.39–1.16 (m, 2H, 4-H), 0.81 (dd, *J* = 7.5, 7.5 Hz, 3H, 5-H); ^13^C NMR (CDCl_3_): δ 172.7, 170.2, 138.6, 138.5, 128.8, 128.8, 128.5, 128.4, 127.2, 127.1, 71.6, 61.2, 56.7, 32.6, 32.1, 18.5, 13.4; HR MS: calcd for C_21_H_25_NO_4_Na [M + Na]^+^ 378.1676, found 378.1689.

*(R)-2-(Diphenylacetyloxy)-N-methoxy-N,3-dimethylbutanamide ((R)-***6d***).* HPLC (CHIRALPAK IC, *i*-PrOH/hexane = 1/9, flow rate = 1.0 mL/min): *t*_R_ = 11.3 min (17.1%), *t*_R_ = 32.5 min (82.9%); IR (neat): 1735, 1674, 1496, 750, 700 cm^–1^; ^1^H NMR (CDCl_3_): δ 7.42–7.21 (m, 10H, Ph), 5.17 (s, 1H, 2′-H), 5.17 (d, *J* = 6.5 Hz, 1H, 2-H), 3.79 (s, 3H, OMe), 3.22 (s, 3H, NMe), 2.17 (dqq, *J* = 7.0, 6.5, 6.5 Hz, 1H, 3-H), 0.88 (d, *J* = 6.5 Hz, 3H, 4-H), 0.87 (d, *J* = 6.5 Hz, 3H, 4-H); ^13^C NMR (CDCl_3_): δ 172.6, 169.5, 138.6, 138.5, 128.8, 128.8, 128.6, 128.3, 127.2, 127.0, 75.8, 61.1, 56.9, 32.0, 29.9, 18.7, 17.3; HR MS: calcd for C_21_H_25_NO_4_Na [M + Na]^+^ 378.1676, found 378.1686.

*(R)-2-(Diphenylacetyloxy)-N-methoxy-N-methylhexanamide ((R)-***6e***).* HPLC (CHIRALPAK IC, *i*-PrOH/hexane = 1/9, flow rate = 1.0 mL/min): *t*_R_ = 10.4 min (1.8%), *t*_R_ = 25.4 min (98.2%); IR (neat): 1736, 1678, 1498, 1445, 743, 704 cm^–1^; ^1^H NMR (CDCl_3_): δ 7.35–7.17 (m, 10H, Ph), 5.27 (dd, *J* = 8.5, 4.0 Hz, 1H, 2-H), 5.13 (s, 1H, 2-H), 3.73 (s, 3H, OMe), 3.16 (s, 3H, NMe), 1.80–1.67 (m, 2H, 3-H), 1.29–1.13 (m, 4H, 4-H, 5-H), 0.79 (ddd, *J* = 7.0, 7.0, 2.5 Hz, 3H, 6-H); ^13^C NMR (CDCl_3_): δ 172.6, 170.2, 138.6, 138.5, 128.8, 128.8, 128.6, 128.4, 127.2, 127.1, 71.7, 61.2, 56.7, 21.1, 30.2, 27.2, 22.0, 13.7; HR MS: calcd for C_22_H_27_NO_4_Na [M + Na]^+^ 392.1832, found 392.1848.

*(R)-2-(Diphenylacetyloxy)-N-methoxy-N,4-dimethylpentanamide ((R)-***6f***).* HPLC (CHIRALPAK IC, *i*-PrOH/hexane = 1/9, flow rate = 1.0 mL/min): *t*_R_ = 10.1 min (1.8%), *t*_R_ = 24.7 min (98.2%); IR (neat): 1733, 1678, 1491, 752, 702 cm^–1^; ^1^H NMR (CDCl_3_): δ 7.34–7.17 (m, 10H, Ph), 5.31 (dd, *J* = 10.3, 3.0 Hz, 1H, 2-H), 5.12 (s, 1H, 2′-H), 3.74 (s, 3H, OMe), 3.15 (s, 3H, NMe), 1.73 (ddd, *J* = 14.0, 10.0, 4.0 Hz, 1H, 3-H), 1.58–1.48 (m, 1H, 4-H), 1.45 (ddd, *J* = 14.0, 9.5, 3.5 Hz, 1H, 3-H), 0.80 (d, *J* = 6.0 Hz, 3H, 5-H), 0.76 (d, *J* = 6.5 Hz, 5-H); ^13^C NMR (CDCl_3_): δ 172.7, 170.6, 138.5, 138.5, 128.8, 128.8, 128.6, 128.4, 127.2, 127.1, 70.6, 61.2, 56.7, 39.2, 32.2, 24.5, 23.1, 21.1; HR MS: calcd for C_22_H_27_NO_4_Na [M + Na]^+^ 392.1832, found 392.1847.

*(R)-2-Cyclohexyl-2-(diphenylacetyloxy)-N-methoxy-N-methylacetamide ((R)-***6g***).* HPLC (CHIRALPAK IC, *i*-PrOH/hexane = 1/9, flow rate = 1.0 mL/min): *t*_R_ = 10.3 min (20.4%), *t*_R_ = 40.0 min (79.6%); IR (neat): 1736, 1672, 1495, 1451, 752, 700 cm^–1^; ^1^H NMR (CDCl_3_): δ 7.35–7.16 (m, 10H, Ph), 5.13 (d, *J* = 6.5 Hz, 1H, 2-H), 5.11 (s, 1H, 2′-H), 3.75 (s, 3H, OMe), 3.17 (s, 3H, NMe), 1.84–1.75 (m, 1H, *c*-Hex), 1.68–1.41 (m, 5H, *c*-Hex), 1.24–0.92 (m, 5H, *c*-Hex); ^13^C NMR (CDCl_3_): δ 172.6, 169.5, 138.6, 138.5, 128.8, 128.6, 128.3, 127.2, 127.0, 75.4, 61.1, 56.9, 39.3, 31.9, 28.7, 27.8, 26.0, 25.7; HR MS: calcd for C_24_H_29_NO_4_Na [M + Na]^+^ 418.1989, found 418.2003.

*(R)-3-(tert-Butyldimethylsiloxy)-2-(diphenylacetyloxy)-N-methoxy-N-methylpropanamide ((R)-***6i***).* HPLC (CHIRALPAK IC, *i*-PrOH/hexane = 1/9, flow rate = 0.5 mL/min): *t*_R_ = 13.7 min (93.0%), *t*_R_ = 21.2 min (7.0%); IR (neat): 1741, 1670, 1496, 1469, 737, 699 cm^–1^; ^1^H NMR (CDCl_3_): δ 7.36–7.21 (m, 10H, Ph), 5.48 (dd, *J* = 7.0, 4.0 Hz, 1H, 2-H), 5.17 (s, 1H, 2′-H), 3.91 (dd, *J* = 11.0, 4.0 Hz, 1H, 3-H), 3.88 (dd, *J* = 11.0, 7.0 Hz, 1H, 3-H), 3.80 (s, 3H, OMe), 3.20 (s, 3H, NMe), 0.83 (s, 9H, TBS), –0.00 (s, 3H, TBS), –0.03 (s, 3H, TBS); ^13^C NMR (CDCl_3_): δ 172.6, 167.7, 138.5, 138.4, 128.8, 128.8, 128.6, 128.4, 127.2, 127.1, 73.3, 62.0, 61.3, 56.7, 32.1, 25.7, 18.2, −5.5, −5.6; HR MS: calcd for C_25_H_35_NO_5_SiNa [M + Na]^+^ 480.2177, found 480.2174.

*(R)-4-(tert-Butyldimethylsiloxy)-2-(diphenylacetyloxy)-N-methoxy-N-methylbutanamide ((R)-***6j***).* HPLC (CHIRALPAK IC, *i*-PrOH/hexane = 1/9, flow rate = 1.0 mL/min): *t*_R_ = 9.8 min (2.7%), *t*_R_ = 23.4 min (97.3%); IR (neat): 1738, 1673, 1496, 1469, 762, 701 cm^–1^; ^1^H NMR (CDCl_3_): δ 7.38–7.21 (m, 10H, Ph), 5.25 (d, *J* = 8.5 Hz, 1H, 2-H), 5.16 (s, 1H, 2′-H), 3.78 (s, 3H, OMe), 3.59 (ddd, *J* = 10.0, 6.0, 4.0 Hz, 1H, 4-H), 3.50 (ddd, 10.0, 10.0, 5.0 Hz, 1H, 4’-H), 3.21 (s, 3H, NMe), 2.04–1.84 (m, 2H, 3-H), 0.85 (s, 9H, TBS), –0.03 (s, 3H, TBS), –0.07 (s, 3H, TBS); ^13^C NMR (CDCl_3_): δ 172.5, 170.4, 138.6, 138.5, 128.8, 128.8, 128.6, 128.4, 127.2, 127.1, 68.7, 61.2, 58.3, 56.8, 33.6, 32.2, 25.8, 18.1, −5.5, −5.6; HR MS: calcd for C_26_H_37_NO_5_SiNa (M + Na^+^) 494.2333, found 494.2321.

*(R)-4-(tert-Butyldimethylsiloxy)-2-(diphenylacetyloxy)-N-methoxy-N-methylpentanamide ((R)-***6k***).* HPLC (CHIRALPAK IC, *i*-PrOH/hexane = 1/9, flow rate = 1.0 mL/min): *t*_R_ = 8.8 min (3.0%), *t*_R_ = 21.4 min (97.0%); IR (neat): 1739, 1680, 1496, 1469, 735, 701 cm^–1^; ^1^H NMR (CDCl_3_): δ 7.39–7.20 (m, 10H, Ph), 5.34 (dd, *J* = 8.5, 4.5 Hz, 1H, 2-H), 5.16 (s, 1H, 2′-H), 3.77 (s, 3H, OMe), 3.54 (t, *J* = 6.0 Hz, 2H, 5-H), 3.20 (s, 3H, NMe), 1.93–1.77 (m, 2H, 3-H), 1.58–1.42 (m, 2H, 4-H), 0.87 (s, 9H, TBS), 0.01 (s, 6H, TBS); ^13^C NMR (CDCl_3_): δ 172.6, 170.1, 138.6, 138.5, 128.8, 128.8, 128.6, 128.4, 127.2, 127.1, 71.8, 62.3, 61.2, 56.7, 32.1, 28.4, 27.2, 25.9, 18.2, −5.4; HR MS: calcd for C_27_H_39_NO_5_SiNa [M + Na]^+^ 508.2490, found 508.2514.

The ^1^H and ^13^C-NMR spectra of the compounds are available in Supplementary Materials.

## 4. Conclusions

In summary, we developed an efficient method for producing optically active 2-hydroxyamides based on the KR of racemic 2-hydroxyamides with diphenylacetyl components using (*R*)-BTM as a nucleophilic chiral acyl-transfer catalyst. The resulting chiral compounds could be converted into the other useful chiral compounds without erosion of the chirality. The transition states were determined by DFT calculations to support the observations in their process. Further research on the present method, including the application of this novel protocol to the production of other chiral materials, is currently underway in our laboratory.

## Figures and Tables

**Table 1:**
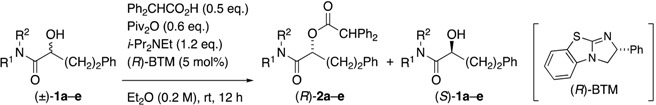
Kinetic resolution (KR) of various racemic 2-hydroxyamides ((±)-**1a**–**1e**).

Entry	R^1^, R^2^	Yield (2; 1) [%]	ee (2; 1) [%]	*s*
1	Me, H (**a**)	42; 52	12; 14	1
2	Bn, H (**b**)	50; 50	17; 15	2
3	Ph, H (**c**)	55; 45	79; 73	18
4 ^a^	Me, Me (**d**)	48; 46	92; >99	254
5 ^a^	Me, OMe (**e**)	50; 50	94; 99	156
6 ^b^	Me, Me (**f**)	47; 50	91; 98	95
7 ^c^	Me, OMe (**g**)	47; 42	94; 98	157

^a^ Ph_2_CHCO_2_H (0.75 eq.), Piv_2_O (0.9 eq.), and *i*-Pr_2_NEt (1.2 eq.) were used. ^b^ (Ph_2_CHCO)_2_O (DPHAA; 0.60 eq.) and *i*-Pr_2_NEt (0.60 eq.) were used. ^c^ (Ph_2_CHCO)_2_O (DPHAA; 0.75 eq.) and *i*-Pr_2_NEt (0.75 eq.) were used.

**Table 2:**
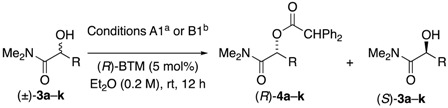
KR of 2-hydroxy dimethylamide ((±)-**3a**–**3k**).

Entry	Substrate	Conditions ^a,b^	Yield (4; 3) [%]	ee (4; 3) [%]	*s*
1	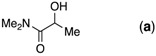	A1	52; 46	89; 82	42
2		B1	37; 56	88; 57	27
3	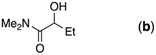	A1	40; 53	93; 54	45
4		B1	37; 54	88; 62	29
5	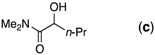	A1	41; 57	94; 65	65
6		B1	45; 47	93; 74	63
7	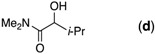	A1	13; 76	82; 14	12
8		B1	25; 75	63; 21	5
9	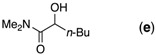	A1	41; 56	95; 69	75
10		B1	36; 60	95; 74	63
11	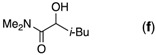	A1	47; 52	95; 78	92
12		B1	48; 45	97; 94	208
13	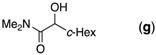	A1	7; 83	66; 6	5
14		B1	43; 49	74; 68	13
15	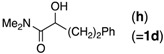	A1	58; 46	92; >99	254
16		B1	47; 50	91; 98	95
17	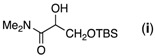	A1	34; 66	82; 41	15
18		B1	45; 49	80; 69	18
19	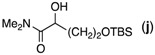	A1	46; 50	93; 95	103
20		B1	44; 51	94; 79	81
21	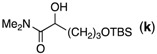	A1	48; 51	94; 99	151
22		B1	47; 46	96; 92	176

^a^ Conditions A1; Ph_2_CHCO_2_H (0.75 eq.), Piv_2_O (0.9 eq.), and *i*-Pr_2_NEt (1.2 eq.). ^b^ Conditions B1; (Ph_2_CHCO)_2_O (DPHAA; 0.60 eq.) and *i*-Pr_2_NEt (0.60 eq.).

**Table 3:**
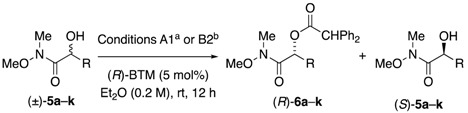
KR of 2-hydroxy-Weinreb amide ((±)-**5a**–**5k**).

Entry	Substrate	Conditions ^a,b^	Yield (6; 5) [%]	ee (6; 5) [%]	*s*
1	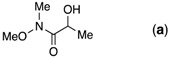	A1	51; 44	93; 99	130
2		B2	50; 50	85; 98	57
3	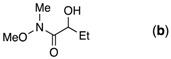	A1	46; 51	96; 85	118
4		B2	46; 49	90; 89	57
5	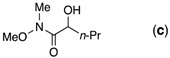	A1	45; 55	96; 94	176
6		B2	45; 42	94; 96	118
7	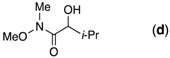	A1	9; 91	66; 7	5
8		B2	27; 65	67; 31	7
9	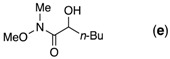	A1	43; 56	96; 69	113
10		B2	49; 50	91; 97	89
11	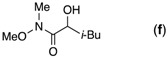	A1	46; 49	97; 89	168
12		B2	46; 44	94; 99	168
13	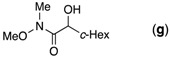	A1	4; 91	59; 5	4
14		B2	35; 61	42; 24	3
15	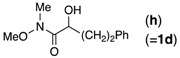	A1	50; 50	94; 99	156
16		B2	47; 42	94; 98	157
17	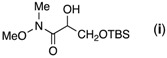	A1	40; 60	86; 51	22
18		B2	50; 46	73; 84	16
19	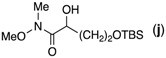	A1	47; 50	95; 91	115
20		B2	49; 48	90; 99	106
21	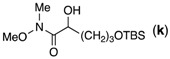	A1	54; 46	94; 99	118
22		B2	48; 41	87; 99	76

^a^ Conditions A1; Ph_2_CHCO_2_H (0.75 eq.), Piv_2_O (0.9 eq.), and *i*-Pr_2_NEt (1.2 eq.). ^b^ Conditions B2; (Ph_2_CHCO)_2_O (DPHAA; 0.75 eq.) and *i*-Pr_2_NEt (0.75 eq.).

## Supplementary Materials

The Supplementary Materials containing 1H and 13C NMR spectroscopic data are available online, Figure S1: Preferable transition structure ((R)-3a-TS), Table S1: Cartesian Coordinates (Angstroms), Figure S2: Unfavorable transition structure ((S)-3a-TS), Table S2: Cartesian Coordinates (Angstroms), Figure S3: Preferable transition structure ((R)-5a-TS), Table S3: Cartesian Coordinates (Angstroms), Figure S4: Unfavorable transition structure ((S)-5a-TS), Table S4: Cartesian Coordinates (Angstroms).
