# Supplementary material for: Kinetic Resolution of Racemic 2-Hydroxyamides Using a Diphenylacetyl Component as an Acyl Source and a Chiral Acyl-Transfer Catalyst
Source: Molecules. 2018 Aug 10;23(8):2003. doi: 10.3390/molecules23082003 (PMC6222459; doi:10.3390/molecules23082003)
Supplement: Supplementary file 1 [file molecules-23-02003-s001.pdf]

Molecules **2018**, *23*, 2003; doi:10.3390/molecules23082003 www.mdpi.com/journal/molecules

Supplementary Materials: Kinetic Resolution of Racemic 2-Hydroxyamides Using a Diphenylacetyl Component as an Acyl Source and a Chiral Acyl-Transfer Catalyst

Takatsugu Murata, Tatsuya Kawanishi, Akihiro Sekiguchi, Ryo Ishikawa, Keisuke Ono, Kenya Nakata and Isamu Shiina

Cartesian Coordinates of ((*R*)-**3a-TS**), ((*S*)-**3a-TS**), ((*R*)-**5a-TS**), ((*S*)-**5a-TS**)
^{1}H and ^{13}C NMR Spectroscopic Data of Compounds
Copy of HPLC analyses of Compounds

(Cartesian Coordinates of (S)-**3a-TS**, (R)-**3a-TS**, (S)-**5a-TS** and (R)-**5a-TS**)

All calculations were performed with the program package Spartan *'10* 1.1.0 of Wavefunction Inc. (http://www.wavefun.com). All structures were optimized and subjected to frequency analysis with the B3LYP/6-31G* method, followed by single point calculations to provide the thermodynamic properties.

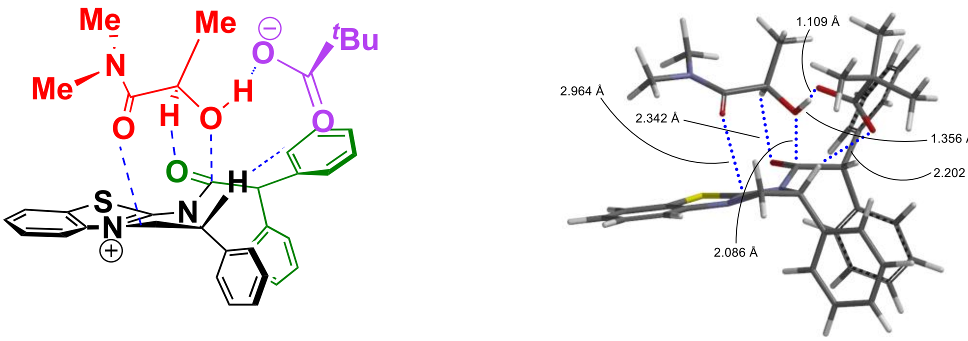

Figure S1. Preferable transition structure ((*R*)-**3a-TS**).
E(B3LYP/6-31G*) = –2450.63466 au
ν_{TS} = 258*i* cm^{-1}

Table S1. Cartesian Coordinates (Angstroms)

Atom	*X*	*Y*	*Z*
C	0.276033	0.407742	1.269097
C	1.797699	0.498867	1.156432
H	2.073283	0.217362	0.138101
O	−0.340336	0.410035	2.333515
N	−0.466607	0.949403	0.123524
C	−1.782791	1.092369	0.208050
N	−2.395278	1.122479	−0.984420
C	−1.458850	0.670358	−2.026805
C	−0.080252	0.885098	−1.323416
C	0.643398	2.130599	−1.804712

C	1.693502	1.979696	−2.719048
C	2.328325	3.103160	−3.251763
C	1.927775	4.383752	−2.869534
C	0.892555	4.538368	−1.944933
C	0.252535	3.418201	−1.416885
H	−0.534512	3.549662	−0.679294
H	0.587800	5.532156	−1.627942
H	2.426533	5.257955	−3.279673
H	3.145241	2.973968	−3.956839
H	2.026439	0.980438	−2.986031
H	0.554396	0.012257	−1.467842
H	−1.653258	−0.387735	−2.221687
H	−1.553505	1.272820	−2.931429
O	0.165337	−1.522964	0.492982
C	−0.644206	−2.320415	1.283339
C	−2.116792	−2.266667	0.784289
N	−3.110375	−2.782827	1.583094
O	−2.390759	−1.761886	−0.309324
H	−0.630343	−1.914090	2.308680
C	−0.113202	−3.771634	1.313511
H	0.926691	−3.747488	1.652302
H	−0.682650	−4.425860	1.982661
H	−0.135502	−4.203962	0.308213
H	0.427653	−2.083652	−0.713887
O	0.636715	−2.642349	−1.648839
C	1.710637	−2.232619	−2.274664
C	2.161429	−3.169965	−3.412358
O	2.319666	−1.191281	−2.001109
S	−2.853343	1.238951	1.558084
C	−3.781397	1.211298	−0.921692
C	−4.219366	1.330363	0.410049
C	−5.573899	1.456824	0.701878
H	−5.916015	1.547375	1.728597
C	−4.685780	1.212470	−1.981923
H	−4.341315	1.114814	−3.006311
C	−6.042179	1.334824	−1.681714
H	−6.766176	1.333523	−2.490921
C	−6.483135	1.459960	−0.358167
H	−7.544180	1.559102	−0.150565
C	0.981571	−3.383419	−4.384091
H	1.271236	−4.079454	−5.180921
H	0.681809	−2.439659	−4.856739
H	0.114615	−3.792886	−3.858752
C	2.561830	−4.524257	−2.786373
H	2.864607	−5.227632	−3.571798
H	1.726096	−4.959964	−2.231533
H	3.405674	−4.404022	−2.096685
C	3.358132	−2.559301	−4.155701
H	3.702225	−3.242651	−4.941638
H	4.190775	−2.365761	−3.473066

H	3.089496	−1.606814	−4.624459
C	2.500581	−0.484685	2.091980
C	3.862480	−2.302976	3.748426
C	3.267759	−1.517163	1.538466
C	2.427051	−0.370359	3.485626
C	3.099686	−1.276013	4.307132
C	3.946288	−2.418667	2.359794
H	3.319091	−1.615476	0.457726
H	1.839422	0.424631	3.931618
H	3.029076	−1.175444	5.387566
H	4.539211	−3.212964	1.912678
H	4.389818	−3.004799	4.389814
C	2.275377	1.939352	1.392772
C	3.298100	4.528971	1.819554
C	1.647588	2.818403	2.285653
C	3.420627	2.380852	0.714756
C	3.930128	3.660568	0.926995
C	2.155107	4.102856	2.495282
H	0.757227	2.497192	2.817003
H	3.917334	1.712497	0.016082
H	4.819545	3.980679	0.390276
H	1.653018	4.769634	3.192204
H	3.692964	5.528109	1.984812
C	−2.934946	−3.291896	2.933649
H	−1.949331	−3.048055	3.323924
H	−3.069249	−4.382279	2.972372
H	−3.683212	−2.836106	3.595484
C	−4.480166	−2.800593	1.092722
H	−5.121220	−2.148197	1.703320
H	−4.888960	−3.819170	1.134579
H	−4.482894	−2.445006	0.063444

Requested basis set is 6-31G(d)
There are 279 shells and 810 basis functions

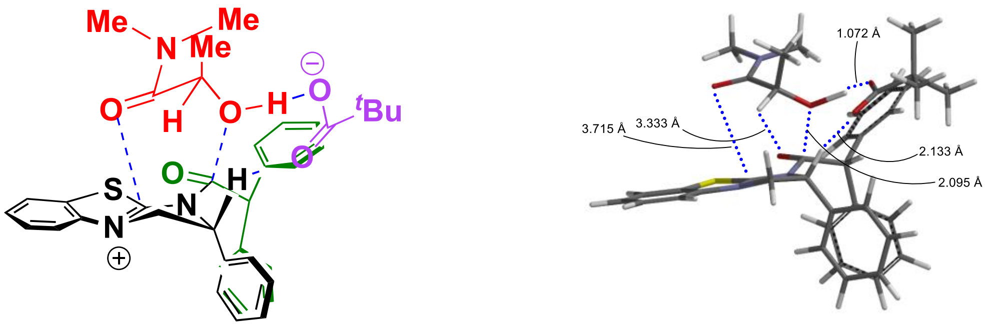

Figure S2. Unfavorable transition structure ((*S*)-**3a**-**TS**).
E(B3LYP/6-31G*) = –2450.62825 au
ν_{TS} = 95*i* cm^{-1}

Table S2. Cartesian Coordinates (Angstroms)

Atom	*X*	*Y*	*Z*
C	−0.207745	−0.302275	1.069726
C	1.166811	0.272135	1.426100
H	1.640542	0.613352	0.502855
O	−0.856159	−1.008563	1.836484
N	−1.038343	0.496332	0.148310
C	−2.342133	0.229429	0.080987
N	−2.934700	0.743126	−1.011597
C	−1.932567	1.272381	−1.941200
C	−0.634697	1.293053	−1.061223
C	−0.198170	2.711908	−0.751493
C	0.877193	3.254210	−1.465617
C	1.250669	4.585579	−1.268936
C	0.556788	5.382589	−0.358226
C	−0.516007	4.844568	0.356610
C	−0.895538	3.518866	0.156503
H	−1.719415	3.104802	0.731798
H	−1.054835	5.455813	1.075274
H	0.851105	6.416991	−0.202013
H	2.087047	4.997098	−1.827984
H	1.408801	2.629665	−2.178802
H	0.157886	0.748861	−1.576908
H	−1.837442	0.605455	−2.804825
H	−2.205654	2.273937	−2.278968
O	0.556780	−1.473989	−0.489881
C	−0.413027	−2.250796	−1.122715
H	1.741191	−1.179031	−1.258294
O	2.689909	−1.016151	−1.730246
C	2.624762	−0.191519	−2.754828
C	3.970656	−0.004377	−3.478089
O	1.596850	0.389059	-3.109370
S	−3.410236	−0.646372	1.114183
C	−4.296343	0.472328	−1.118908
C	−4.744177	−0.265734	−0.008135
C	−6.079750	−0.644167	0.092818
H	−6.429468	-1.223770	0.941825
C	−5.165468	0.847764	−2.141853
H	−4.811494	1.415198	−2.997017
C	−6.502315	0.466305	−2.030303
H	−7.199312	0.743625	−2.815337
C	−6.955950	−0.268511	−0.927139
H	−8.001180	−0.555305	−0.863988
C	4.492743	−1.385949	−3.927485
H	5.457341	−1.276951	−4.438050
H	3.794953	−1.863443	−4.625695
H	4.626108	−2.053139	−3.071566
C	4.973770	0.628028	−2.487617
H	5.942543	0.776183	−2.979671

H	5.125945	−0.014693	−1.615632
H	4.624613	1.607068	−2.136196
C	3.782675	0.914200	-4.694392
H	4.738250	1.050005	−5.214707
H	3.411342	1.899595	−4.395893
H	3.061062	0.493185	−5.401482
H	−1.286922	−1.635721	−1.408502
C	0.106351	−2.878725	−2.433757
H	0.388247	−2.085896	−3.134242
H	−0.676971	−3.490969	−2.894701
H	0.983242	−3.509218	−2.254573
C	1.030376	1.489837	2.353637
C	0.937185	3.724935	4.064779
C	0.045415	1.590102	3.345669
C	1.967102	2.525392	2.235720
C	1.924101	3.631988	3.081557
C	−0.000865	2.700539	4.191589
H	−0.691555	0.800784	3.456158
H	2.737764	2.462384	1.471650
H	2.661262	4.423048	2.970433
H	−0.773727	2.759802	4.954149
H	0.901019	4.587093	4.725728
C	2.087840	−0.771128	2.063474
C	3.882719	−2.576937	3.266096
C	3.333015	−1.037549	1.483177
C	1.754272	−1.418635	3.261441
C	2.641744	−2.317461	3.852695
C	4.224718	−1.932537	2.077746
H	3.598702	−0.563259	0.543821
H	0.794009	−1.228868	3.725820
H	2.364022	−2.811651	4.780549
H	5.184588	−2.126361	1.605605
H	4.574939	−3.273470	3.732327
C	−1.094660	−3.352881	−0.265317
O	−2.324158	−3.460911	−0.348232
N	−0.344580	−4.207497	0.491390
C	1.106278	−4.193380	0.605032
H	1.509233	−3.289301	0.157494
H	1.541286	−5.084051	0.127188
H	1.391762	−4.200307	1.664073
C	−1.018251	−5.254937	1.241243
H	−0.673863	−6.246464	0.914453
H	−2.090408	−5.167802	1.070607
H	−0.802300	−5.153435	2.313361

Requested basis set is 6-31G(d)

There are 279 shells and 810 basis functions

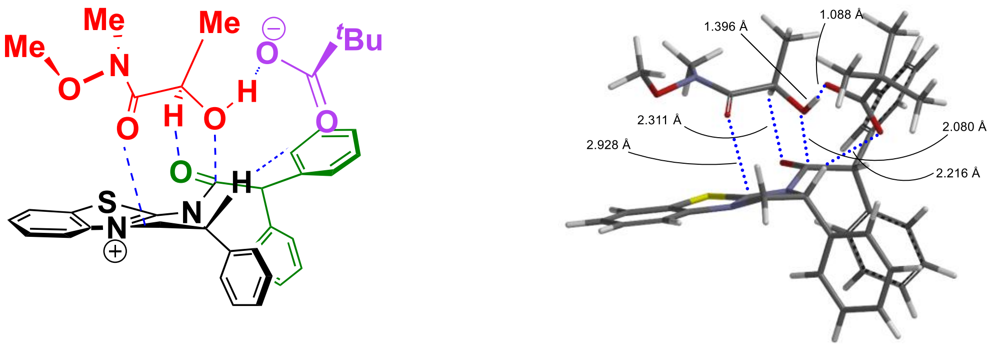

Figure S3. Preferable transition structure ((*R*)-**5a-TS**).
E(B3LYP/6-31G*) = –2525.79242 au
ν_{TS} = 172*i* cm^{-1}

Table S3. Cartesian Coordinates (Angstroms)

Atom	*X*	*Y*	*Z*
C	0.387394	0.470816	1.235218
C	1.913829	0.456259	1.149743
H	2.185890	0.125098	0.145199
O	−0.245390	0.567904	2.286722
N	−0.298397	1.014262	0.055040
C	−1.600228	1.268461	0.112971
N	−2.188665	1.318656	−1.090555
C	−1.277440	0.777680	−2.110478
C	0.103239	0.868320	−1.381424
C	0.953396	2.020432	−1.887746
C	2.001885	1.740216	−2.772773
C	2.746953	2.781207	−3.330445
C	2.457918	4.106320	−3.003478
C	1.424055	4.388937	−2.107964
C	0.674096	3.351886	−1.555642
H	−0.114834	3.581748	−0.844640
H	1.204983	5.417317	−1.833690
H	3.042327	4.915464	−3.433544
H	3.561931	2.552440	−4.012158
H	2.246780	0.705826	−2.997224
H	0.648793	−0.068567	−1.485436
H	−1.566478	−0.256894	−2.312282
H	−1.296739	1.383922	−3.017363
O	0.173592	−1.486172	0.564648
C	−0.740868	−2.147900	1.359971
C	−2.176412	−1.948354	0.794157
N	−3.242490	−2.306546	1.604215
O	−2.375510	−1.518769	−0.338655
H	−0.726970	−1.691443	2.363984
C	−0.390151	−3.646119	1.490037
H	0.628698	−3.726874	1.879919
H	−1.068776	−4.184780	2.161887
H	−0.421377	−4.131398	0.509400

H	0.440708	−2.172842	−0.621306
O	0.642692	−2.809397	−1.480722
C	1.723706	−2.461881	−2.136010
C	2.152925	−3.485889	−3.203446
O	2.350244	−1.416794	−1.932662
S	−2.681138	1.528999	1.437405
C	−3.564567	1.521967	−1.057115
C	−4.015344	1.699428	0.262714
C	−5.360222	1.935726	0.525809
H	−5.713904	2.064347	1.544147
C	−4.444673	1.577309	−2.135898
H	−4.089566	1.435147	−3.151477
C	−5.791966	1.813602	−1.865043
H	−6.498467	1.857776	−2.688438
C	−6.245790	1.994488	−0.552264
H	−7.299570	2.179031	−0.367030
C	0.963517	−3.752546	−4.149905
H	1.238764	−4.502840	−4.901286
H	0.669172	−2.839093	−4.681486
H	0.096085	−4.116669	−3.593034
C	2.541189	−4.794465	−2.479398
H	2.833814	−5.559008	−3.209417
H	1.704273	−5.178914	−1.889298
H	3.389291	−4.632621	−1.803372
C	3.352577	−2.946147	−3.995446
H	3.692153	−3.693617	−4.722755
H	4.187441	−2.700988	−3.332366
H	3.089393	−2.033757	−4.541285
C	2.534381	−0.540491	2.128821
C	3.754135	−2.381322	3.869276
C	3.277194	−1.614962	1.624376
C	2.413751	−0.395488	3.516417
C	3.015369	−1.312416	4.379467
C	3.884457	−2.528172	2.487272
H	3.362185	−1.739324	0.548420
H	1.841214	0.430297	3.924062
H	2.908055	−1.187881	5.454358
H	4.457973	−3.356436	2.078060
H	4.226514	−3.092059	4.542890
C	2.484742	1.868002	1.352354
C	3.673453	4.395015	1.722078
C	1.908911	2.810406	2.215145
C	3.662520	2.214583	0.675446
C	4.253871	3.463071	0.859098
C	2.498824	4.063478	2.396646
H	0.994258	2.563325	2.744613
H	4.119688	1.497015	−0.000993
H	5.167426	3.708822	0.323648
H	2.035845	4.780134	3.070769
H	4.132498	5.369842	1.865327

C	−3.221552	−2.361901	3.057797
H	−2.283878	−2.799041	3.398355
H	−4.045974	−2.996982	3.393367
H	−3.336625	−1.363237	3.499320
O	−4.494392	−1.801067	1.178685
C	−5.121154	−2.717467	0.280226
H	−4.532548	−2.826389	−0.634912
H	−6.089863	−2.266031	0.047625
H	−5.272884	−3.696619	0.752558

Requested basis set is 6-31G(d)
There are 283 shells and 825 basis functions

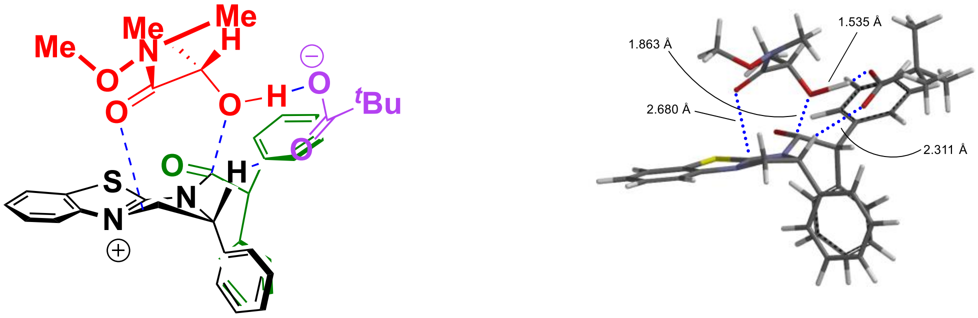

Figure S4. Unfavorable transition structure ((*S*)-**5a-TS**).
E(B3LYP/6-31G*) = –2525.78725 au
ν_{TS} = 136*i* cm^{-1}

Table S4. Cartesian Coordinates (Angstroms)

Atom	*X*	*Y*	*Z*
C	0.006980	0.812077	1.144269
C	1.328094	1.482134	0.717279
H	1.691501	0.966951	−0.176236
O	−0.577688	1.135441	2.186467
N	−0.950485	0.600978	−0.002543
C	−2.249553	0.579353	0.249458
N	−2.978080	0.021585	−0.734241
C	−2.091522	−0.679788	−1.671787
C	−0.706237	−0.024344	−1.345554
C	−0.288717	0.974466	−2.412901
C	0.741252	0.628540	−3.296652
C	1.092754	1.490398	−4.338328
C	0.422206	2.701994	−4.505193
C	−0.602725	3.053241	−3.623481
C	−0.957103	2.194337	−2.584855
H	−1.740191	2.491740	−1.892664
H	−1.121125	4.001214	−3.738474
H	0.699478	3.373540	−5.313619
H	1.895061	1.213136	−5.017394
H	1.264998	−0.313391	−3.160309
H	0.062581	−0.787854	−.224766

H	−2.119307	−1.750818	−1.447776
H	−2.388476	−0.494705	−2.705728
O	0.683981	−0.923877	1.169452
C	0.154641	−1.950649	1.985975
H	2.004886	−1.429557	0.572756
O	2.899375	−.869082	0.286354
C	2.911174	−2.176525	−1.001017
C	4.247239	−2.782777	−1.460230
O	1.958840	−1.998317	−1.755756
S	−3.190819	1.134055	1.591116
C	−4.341144	-0.064740	-0.472883
C	−4.655683	0.544964	0.755272
C	−5.971879	0.589484	1.203110
H	−6.220066	1.053752	2.152983
C	−5.328747	−0.640365	−1.269879
H	−5.076441	−1.112894	−2.213893
C	−6.645888	−0.594768	−0.811364
H	−7.432581	−1.040499	−1.412718
C	−6.966348	0.015495	0.407270
H	−7.998769	0.041079	0.742674
C	4.562289	−4.023498	−0.596669
H	5.519722	−4.457825	−0.907288
H	3.792633	−4.795937	−0.715739
H	4.626930	−3.761150	0.462695
C	5.355583	−1.724786	−1.260248
H	6.322396	−2.132707	−1.579192
H	5.431954	−1.429743	−0.209877
H	5.158828	−0.826551	−1.858472
C	4.154597	−3.178548	−2.941212
H	5.105281	−3.613944	−3.270671
H	3.935035	−2.311608	−3.571893
H	3.362344	−3.915857	−3.106173
H	0.952077	−2.697670	2.103693
C	−0.263916	−1.538892	3.401839
H	0.551595	−0.976820	3.864709
H	−0.458518	−2.438616	3.999768
H	−1.158614	−0.918838	3.393820
C	1.111720	2.954725	0.330223
C	0.887603	5.673659	−0.378370
C	0.256950	3.811148	1.038764
C	1.852053	3.485906	−0.735054
C	1.745433	4.830926	−1.086747
C	0.144074	5.156404	0.683052
H	−0.322200	3.418605	1.866734
H	2.516594	2.837548	-1.300610
H	2.330394	5.217946	-1.917267
H	−0.527017	5.802906	1.243586
H	0.800462	6.722616	−0.650221
C	2.429644	1.386425	1.776241
C	4.559895	1.324487	3.621286

C	3.733896	1.087747	1.362760
C	2.209777	1.670712	3.131844
C	3.264969	1.631016	4.043925
C	4.790907	1.054584	2.272845
H	3.926278	0.870621	0.315499
H	1.207459	1.904539	3.469217
H	3.072295	1.846777	5.092190
H	5.792994	0.814958	1.925347
H	5.379238	1.298407	4.335260
C	−1.040722	−2.572203	−.231871
O	−2.144729	−2.028370	1.225063
N	−0.852937	−3.759085	0.554962
C	0.382301	−4.388126	0.114367
H	1.206016	−4.085743	0.757936
H	0.612707	−4.099215	−0.916273
H	0.266586	−5.475049	0.171316
O	−1.888981	−4.098331	−0.343289
C	−2.939244	−4.796754	0.333278
H	−3.439524	−4.139275	1.048671
H	−2.553167	−5.690542	0.838609
H	−3.634743	−5.093231	−0.456665

Requested basis set is 6-31G(d)

There are 283 shells and 825 basis functions
